# Seagrass Tolerance to Simulated Herbivory Along a Latitudinal Gradient: Predicting the Potential Effects of Tropicalisation

**DOI:** 10.1002/ece3.70561

**Published:** 2024-11-17

**Authors:** Ruby G. Garthwin, Alistair G. B. Poore, Giulia Ferretto, Jeffrey T. Wright, Adriana Vergés

**Affiliations:** ^1^ Centre for Marine Science and Innovation, School of Biological, Earth and Environmental Sciences University of New South Wales Sydney New South Wales Australia; ^2^ Sydney Institute of Marine Science Mosman New South Wales Australia; ^3^ School of Biological Sciences & Oceans Institute University of Western Australia Perth Western Australia Australia; ^4^ Institute for Marine and Antarctic Studies University of Tasmania Hobart Tasmania Australia

**Keywords:** climate change, herbivory, productivity, resilience, spatial shift, temperate seagrass, tropicalisation, warming oceans

## Abstract

The polewards range expansion of tropical herbivorous fish into temperate latitudes is leading to overgrazing of marine habitats and community phase shifts in some regions. Here, we test the potential effects of increased herbivory on the temperate habitat‐forming seagrass *Posidonia australis*. We used a series of simulated herbivory experiments to predict the potential impacts of climate‐mediated increases in seagrass consumption along *P. australis* entire latitudinal range (~9° latitude) in eastern Australia (1700 km of coastline). We subjected treatment plots to two levels of simulated herbivory (10% or 80% of leaves clipped) and compared them to unclipped controls. We measured seagrass leaf growth rates and tissue chemical traits: carbohydrates in rhizomes, leaf phenolics, and nutrients (carbon, nitrogen, and C:N ratio) in leaves and rhizomes. At the warmest range‐edge population, we also tested how responses to increased herbivory may vary between summer and winter, or with repeated clipping events. Clipped shoots maintained growth rates similar to unclipped controls despite losing up to 80% of leaf biomass. This was consistent along the full latitudinal range and after repeated simulated herbivory at the northernmost location. One‐off clipping events impacted plant architecture, increasing the number of subdividing shoots. At the species range edge, leaves grew more in winter than in summer, and clipping tended to lower seagrass growth only in winter; however, higher levels of shoot subdivision were produced over summer than in winter. Plant chemical traits could not explain consistently the growth patterns observed despite some traits varying with latitude (e.g., leaf nitrogen content decreased with latitude and C:N ratio increased) and/or simulated herbivory. *Synthesis*: *P. australis* growth is not affected by increases in simulated herbivory and may be relatively resilient to future increases in seagrass consumption, suggesting that this species could be a relative ‘winner’ under future climate change conditions that lead to enhanced herbivory.

## Graphical abstract

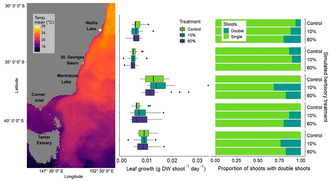

## 1 Introduction

Climate change can profoundly impact species interactions and lead to cascading effects on ecological communities (Blois et al. 2013; Schleuning et al. 2020). In particular, climate‐mediated changes to plant‐herbivore interactions can result in major community level impacts (Burkepile and Parker 2017; Hamann et al. 2020). Herbivores provide a crucial link between primary producers and higher trophic levels and strongly influence the structure and functioning of communities (Mcnaughton et al. 1989; Agrawal and Maron 2022). Climate change may impact existing plant‐herbivore interactions when consumers respond to warming faster than the plants they consume (Corlett and Westcott 2013; Hamann et al. 2020). Shifts in the distributions of consumers can also lead to the development of new interactions and altered ecosystem dynamics (Hamann et al. 2020). Such climate‐mediated changes to species interactions are emerging as an important threat to ecological communities (Vergés et al. 2019) and in many instances can have greater impacts than the direct effects of temperature on individual species (Ockendon et al. 2014).

As temperature varies predictably with latitude, recent studies have sought to understand latitudinal patterns in species interactions to better predict the impacts of global climate change on ecological communities (Longo et al. 2018; Vergés et al. 2018). The strength of biotic interactions such as herbivory, predation and competition often vary with latitude and are commonly perceived to be stronger in the tropics (Roslin et al. 2017; Zvereva and Kozlov 2021). More intense interactions near the tropics, however, may not translate into greater net impacts (Poore et al. 2012). For example, although rates of marine herbivory are often higher in the tropics (Longo et al. 2018), the net impact of herbivores may nevertheless be similar across latitudes if plant productivity matches consumption patterns. This has been shown in some seagrass meadows, where herbivory can have a similar net impact across latitudes because faster growth rates in the tropics compensate for greater consumption rates in these regions (Vergés et al. 2018).

In marine systems, species are shifting their distribution toward the poles faster, on average, than in terrestrial systems (Lenoir et al. 2020). Tropical and warm‐temperate herbivorous fish are among the marine species that are becoming increasingly abundant in higher latitudes with catastrophic consequences for some local communities (Vergés et al. 2014a; Kumagai et al. 2018). The intrusion of tropical herbivores into temperate reefs in the Mediterranean and Australia has led to the overgrazing of algal forests, causing dramatic community phase shifts toward low biomass turf‐dominated systems (Vergés et al. 2014b; Bennett et al. 2015). Tropical herbivores are expected to continue to expand their distribution further into temperate latitudes, potentially leading to increases in herbivory in other marine systems such as seagrass meadows (Hyndes et al. 2016).

Seagrasses are dominant habitat formers along the coastlines of all continents except Antarctica, supporting high biodiversity and providing critical ecosystem services (e.g., fisheries, nutrient cycling and carbon sequestration; Duarte and Krause‐Jensen 2017; De los Santos et al. 2020). The impacts of climate change on seagrass communities can be diverse and complex (Hernán et al. 2017; Nguyen et al. 2021). Ocean warming and extreme temperature events can have strong direct impacts on individual seagrass species, for example marine heatwaves causing the large‐scale die‐back in some Western Australian meadows (Strydom et al. 2020). Seagrass meadows can also be dramatically shaped by grazing (Scott, York, and Rasheed 2021; Valentine and Heck 2021), including by megaherbivores (e.g., dugongs and green turtles; Rodriguez and Heck 2020) and macroherbivores (e.g., fish and urchins; Tomas, Turon, and Romero 2005). However, the effects of warming on seagrass herbivory are less clear, with some studies reporting no effects of warming on seagrass‐herbivore interactions (Garthwin, Poore, and Vergés 2014) or even reduced herbivory on some species (Pagès et al. 2018). Other studies show that warming can intensify herbivory by some common grazers; for example the temperate seagrass *Posidonia oceanica* in the Mediterranean Sea grows more slowly and becomes more palatable in warmer conditions, thus becoming more vulnerable to herbivory by the fish *Sarpa salpa* (Buñuel et al. 2021). These studies have focused on the impacts of warming on existing herbivore‐seagrass interactions, and we know little about the potential effects of novel interactions that may emerge as herbivores continue to expand their ranges and intrude into higher latitudes (Hyndes et al. 2016) but see Santana‐Garcon et al. (2023).

Recent studies suggest that increased tropicalisation could significantly reduce the canopy height and structural complexity of temperate seagrass meadows (forming ‘mowed patches’; Tomas, Turon, and Romero 2005), altering the valuable ecosystem services they provide (e.g., the nursery function for fishery species; Hyndes et al. 2016; Valentine and Heck 2021) due to both greater abundance and diversity of herbivorous fishes and higher consumption rates in the tropics compared to temperate locations (Vergés et al. 2018). In tropical systems, intense grazing on seagrass by parrotfish can remove up to ten times the daily production (Unsworth et al. 2007). There is already evidence of warm‐affiliated herbivores, most likely tropical range extending rabbitfish, selectively grazing on nutritious cool‐edge seagrass transplanted to warm‐edge locations in the eastern Mediterranean Sea (Bennett et al. 2022). Similarly, tropical herbivorous fish are increasing in temperate latitudes along Western Australia and were observed consuming large amounts of the temperate seagrass *Posidonia australis* around Rottnest Island (Michael Tropiano, Pers. Comm, 12 October 2015). Their expansion will likely result in an increased pressure on temperate seagrass meadows (Samsonova 2020; Zarco‐Perello et al. 2020). Impacts of increased herbivory may vary latitudinally, a recent experiment found that negative effects of simulated herbivory (e.g., overgrazing) increase with latitude across the entire latitudinal range of turtlegrass ( *Thalassia testudinum* ) in the western North Atlantic (Campbell et al. 2024).

Whether lower latitude seagrasses can persist under conditions of higher herbivory will be influenced by plant resistance and tolerance to consumers. Seagrasses can resist herbivory by increasing deterrent chemicals such as phenolics in the leaves or tolerate herbivory by inducing compensatory growth or altering chemical contents of the rhizomes (Vergés et al. 2008; Tomas et al. 2015; Valentine and Heck 2021). Changes to plant tissue traits in response to grazing could also influence subsequent palatability of seagrass to herbivores (Buñuel et al. 2021), as nutritional characteristics of seagrass can strongly influence herbivore preference (Vergés et al. 2007; Jiménez‐Ramos et al. 2017). Temperate seagrass may be particularly vulnerable to increased herbivory during the winter months, when light availability and growth rates are lowest (West and Larkum 1979; Dennison 1987; Cambridge and Hocking 1997). It is therefore important to understand the ability of seagrasses to tolerate or compensate for increased herbivory at different latitudes and during different seasons.

In this study, after measuring levels of natural herbivory, we tested the effects of increased herbivory along a latitudinal gradient by experimentally clipping the leaves of a dominant seagrass in eastern Australia, *Posidonia australis* (Hook.f.). There is evidence of some fish species feeding on *Posidonia australis* in this region (e.g., *Meuschenia freycineti* ; Bell et al. 1978), although overall levels of consumption are considered low (Wressnig and Booth 2008). *P. australis* is a large‐leafed, slow‐growing species endemic to temperate Australia that grows mostly in dense, isolated meadows within sheltered bays and estuaries (Gobert et al. 2006). *P. australis* is listed as endangered in some parts of eastern Australia due to widespread losses over the past 50 years, with some populations still declining despite its protection (Evans et al. 2018; West and Glasby 2022). Understanding any potential negative impacts due to climate change is therefore of particular interest for the conservation of this species.

Here, we measured how leaf production varies along the entire latitudinal range of *P. australis* in eastern Australia and used a series of simulated herbivory experiments to predict the potential impacts of climate‐mediated increases in seagrass consumption. Building on previous studies that indicate plant tissue traits may vary with latitude (e.g., leaf nitrogen content generally increases latitudinally; Reich and Oleksyn 2004; Vergés et al. 2018), we also quantified a range of resistance and tolerance tissue chemical traits including leaf total phenolic content, rhizome total non‐structural carbohydrates, and nutrient (carbon and nitrogen) content of the leaves and rhizomes. Specifically, we asked: (1) Does leaf production of *P. australis* vary latitudinally? (2) Is *P. australis* capable of compensating for losses to herbivory via increased growth? (3) Are seagrass responses to herbivory affected by latitude? (4) How do plant tissue chemical traits vary across a latitudinal gradient in response to increased herbivory? And (5) is *P. australis* more vulnerable to increased herbivory during periods of slower growth in the winter months, or (6) after being subjected to repeated herbivory events?

## 2 Materials and Methods

### 2.1 Latitudinal Variation in Leaf Production and Responses to Simulated Herbivory

**FIGURE 1:**
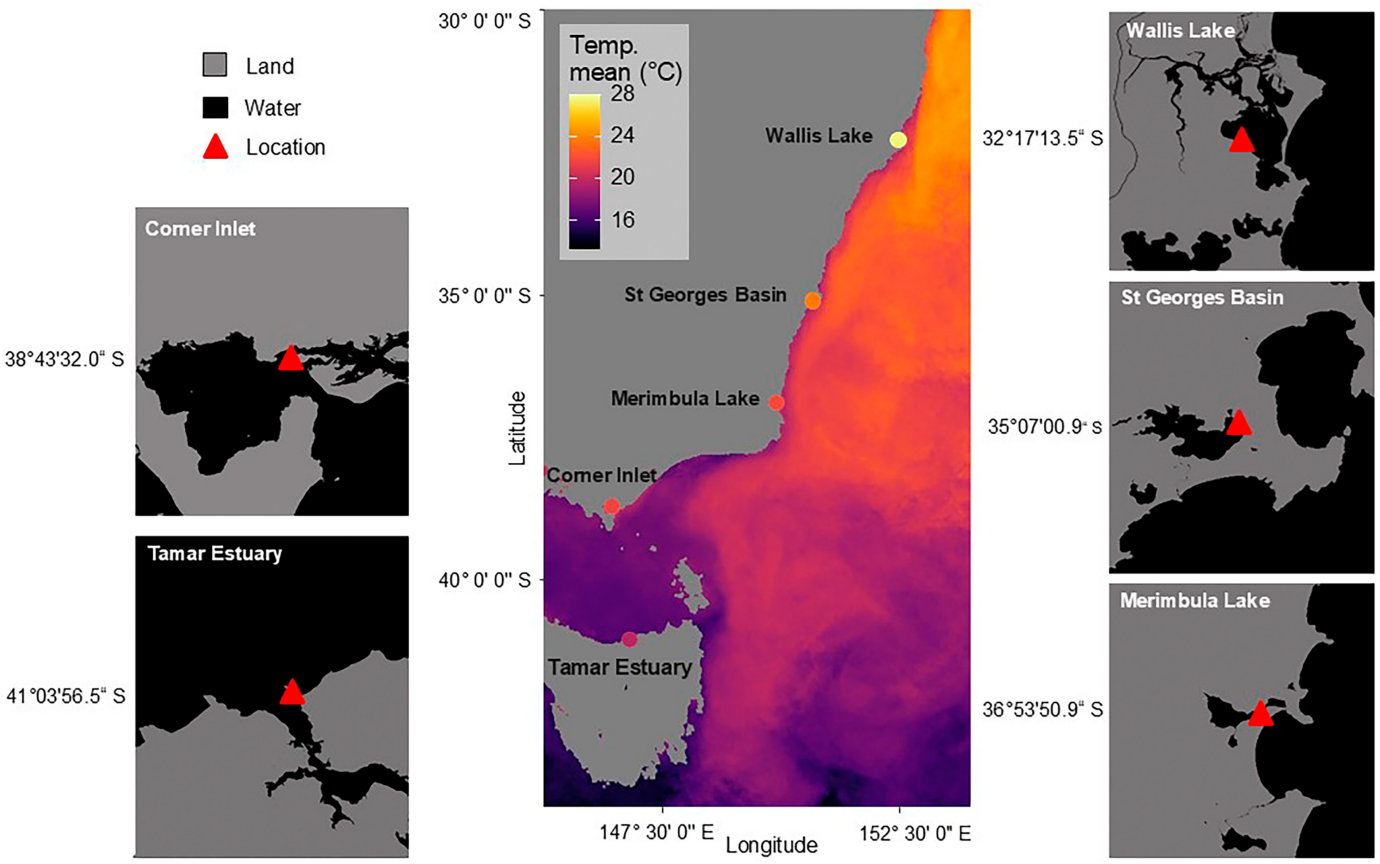
Mean temperature recorded during the 30‐day period for the latitudinal study using loggers (indicated by colored dots over each location) plotted over the average sea surface temperature between December 2015 and February 2016 (Data from the Australian Bureau of Meteorology). Insets show zoomed‐in maps of each estuary.

To determine the impacts of increased grazing on the ability of *P. australis* to compensate for leaf loss, herbivory was simulated along a latitudinal gradient. We also quantified natural levels of leaf production and consumption along the same gradient. We sampled five shallow locations (0.5–3 m depth) covering the entire latitudinal range of *P. australis* in eastern Australia along 1700 km and encompassing 9^o^ of latitude (Figure 1): Wallis lake (32°17′13.5″ S 152°28′09.0″ E), St George's Basin (35°07′00.9″ S 150°39′38.5″ E), Merimbula Lake (36°53′50.9″ S 149°54′30.4″ E), Corner Inlet (38°43′32.0″ S 146°26′06.9″ E) and Tamar Estuary (41°03′56.5″ S 146°47′50.9″ E). *P. australis* in this region occurs mostly at depth between 0.5 and 4 m. Sampling was performed on SCUBA and took place during the austral summer, between November 2015 and February 2016, a time when growth rates were expected to be highest (West and Larkum 1979). All locations were monospecific *P. australis m*eadows except for Corner Inlet, where *Zostera* sp. co‐occurred with *P. australis* . To estimate shoot density, we counted all shoots within five haphazardly placed 0.25 × 0.25 m quadrats at each location.

Continuous patches of *P. australis* of at least 270 m^2^ were initially identified using the high‐quality imagery program NearMap Australia and then validated during fieldwork. Although we aimed to sample in meadows of similar shoot density in each site, this was not possible and there were differences among locations (Figure S1; F4 = 22.67, *p* < 0.001). At each location, we established nine 1.5 m × 1.5 m plots, with plots arranged in a 3 × 3 grid pattern and spaced at least 3 m apart. Treatments were assigned randomly for the first 6 plots, then the remaining 3 were allocated such that the same treatment did not appear twice in any row or column (Figure S2). Three replicate plots were allocated to each of three treatments: Control (shoots un‐manipulated), 10% (removal of 10% of the leaf length, simulating low intensity herbivory) and 80% (removal of 80% of the leaf length, simulating extensive one‐off grazing events; Bennett et al. 2022). The average length of the leaves was variable among sites (Figure S3) and the amount of clipped leaf material was estimated in the field relative to the length of the leaves for that site. For the two levels of simulated herbivory, leaves of all shoots within a plot were cut with scissors, and leaf material was collected in bags and removed from the study area to avoid any effects of detritus accumulation. The leaf clipping procedure has previously been used to mimic the feeding behavior of common macroherbivores (e.g., fish) and results are considered representative of how seagrass will potentially respond to herbivory (Vergés et al. 2008; Sanmartí et al. 2014).

For the duration of the one‐month clipping experiment water temperature was recorded every 60 min at each site using loggers (Onset HOBO Pendant Temperature/Light Data Logger 64 K, accuracy ±0.53°C) placed within the central treatment plot. Each logger was anchored with a vertical steel post pushed into the sediment and held approximately 30 cm above the sediment at the top of the canopy.

#### 2.1.1 Measuring Leaf Production and Natural Herbivory Rates

Production rates of *P. australis* shoots were measured at each location using the needle‐punch method. Ten replicate shoots within each plot were marked using a needle to punch three parallel holes through all leaves just above the ligule (Romero 1989). Tagged shoots were removed one month later, including a 3 cm portion of the rhizome, and cleaned of epiphytes by scraping carefully with a razor blade. Shoots were separated into rhizomes, new leaf growth (young leaves without punch marks plus the length of new tissue below the leaf scars) and older tissue (from the leaf scars to the leaf tip), and the length and width of each leaf section was recorded. Shoots that were subdividing (i.e., shoots still joined at the base but with double leaf sheaths) were counted and labeled as ‘double’ shoots.

All shoot material was oven‐dried at 60°C for 48 h to a constant weight, and the dry weight (g DW) recorded with a balance. Production rates, as leaf area and leaf biomass produced per shoot per day, were calculated by dividing the growth increase by the number of days between tagging and harvesting and then multiplied by the mean shoot density values at each site (mean of 5 quadrats per site) to obtain rates of production per square meter.

To test if natural herbivory varied with latitude, we calculated leaf loss by recording the length and width of each leaf in situ at the start of the experiment on the same 10 shoots that were tagged for productivity within the control plots. After removal of the shoots, area lost to herbivory was estimated visually by measuring the leaf area lost to new grazing scars using a 1‐mm^2^ grid. Due to the low levels on natural herbivory found, no formal analyses were performed on these data.

#### 2.1.2 Tissue Chemical Traits

To test for latitudinal variation and responses to leaf clipping in the nutritional content of *Posidonia australis*, we analyzed tissue traits in three of the shoots used for measuring productivity. The top 2 cm of each vertical rhizome and all newly produced leaf material were used from each shoot to standardize the tissue types tested. All material was oven‐dried at 60°C for 48 h and ground into a fine powder using a *FastPrep*‐24 5G Instrument benchtop homogenizer. Rhizome soluble carbohydrates (sugars) and insoluble carbohydrates (starch) were measured similarly to Sanmartí et al. (2014). Values were expressed as % DW sugar and starch, and values were added together to obtain the total non‐structural carbohydrates (NSC).

To extract sugars, ground rhizome samples were dissolved in 96% (v/v) ethanol, sonicated for 5 min, heated at 80°C for 15 min then centrifuged, and this process was repeated three times. To extract starch, the pellet remaining after this process was dissolved in 0.1 M NaOH then incubated at room temperature for 24 h. Sugar and starch content were determined using an anthrone assay standardized to sucrose, and absorbance was measured spectrophotomically at 626 nm using a microplate reader (SpectraMax 190, Molecular Devices, Sunnyvale, CA). Absorbance values were converted to % DW sugar and starch, and these were added together to obtain the total non‐structural carbohydrates (NSC).

Total phenolic content of the leaves was measured using a modified Folin‐Ciocalteau method (Bolser et al. 1998). 4–4.5 mg of each sample was weighed into 1 mL of MeOH—water (1:1) and incubated for 24 h at 4°C, followed by colorimetric analysis using the Folin‐Ciocalteau reagent and 20% NaCO_3_ solution. Absorbance was measured spectrophotomically at 765 nm using a microplate reader (SpectraMax 190, Molecular Devices, Sunnyvale, CA) and % phenolic content was determined by comparison to a gallic acid standard curve and expressed as gallic acid equivalents (GAE % dry weight). Carbon and nitrogen content (% DW) for both leaves and rhizomes were determined by combustion using a LECO TruSpec Element Analyzer at the Mark Wainwright Analytical Centre at the University of New South Wales.

### 2.2 Temporal Patterns in Leaf Production and Responses to Simulated Herbivory at the Warmest Range‐Edge Population

Temperate seagrass populations at the warm range edge are expected to experience climate change‐mediated increases in herbivory first due to the range expansion of warm‐water herbivores into temperate waters (Hyndes et al. 2016). Warmer‐edge populations may also vary in their susceptibility to herbivory at different times of the year given the role of temperature in regulating plant growth. To determine how leaf production patterns vary with time in these populations, the simulated herbivory experiment was repeated during the austral winter at the northernmost site, Wallis Lake. New plots and treatments were set up in July 2016, with samples being collected after one month and leaf production rates and chemical traits measured as described above.

### 2.3 Responses to Repeated Simulated Herbivory at the Warmest Range‐Edge Population

We tested the ability of *P. australis* to compensate for repeated grazing events, simulating the impacts of an established population of herbivorous fish feeding throughout the winter months. Seagrass leaves in the plots established in July 2016 were repeatedly clipped every 3 weeks for 4 months, from July to November 2016. Shoots were marked for productivity measures during the final clipping event then removed one month later. Leaf production rates and tissue chemical traits were measured as described above.

### 2.4 Statistical Analyses

The effects of latitude and simulated herbivory on the productivity of *P. australis* were tested using linear mixed models with treatment as a fixed factor with three levels (control, 10% and 80% clipped), latitude as a continuous fixed factor and plot and location as random effects. The latitude of each of the 5 locations was included as a continuous factor that reflects the order of the locations from north to south. A random factor of location was also included in the model to account for any dependencies of samples taken at the same location. Hypothesis tests for random effects using parametric bootstrapping were run to check the validity of this, and they found significant effects of plot (*p* < 0.001) and location (*p* < 0.001). Therefore, they were included in all models as random effects to account for the non‐independence of measures within each of these spatial units. Separate analyses were conducted for production rates measured by area (cm^2^ shoot^−1^ day^−1^) and by biomass (g DW shoot^−1^ day^−1^). The effects of latitude and simulated herbivory on production rates were also tested at the square meter scale at each location using the same analysis design. Tissue chemical traits were analyzed with the same fixed and random effects. The presence or absence of sub‐dividing or ‘double’ shoots were analyzed using a generalized linear mixed effects model (GLMM) with the same design as for productivity, with family as binomial. The effects of season/time and simulated herbivory on leaf production rates and tissue chemical traits were tested using linear mixed models with ‘time’ and ‘treatment’ as fixed factors and ‘plot’ as a random factor. The effects of time and treatment on the presence or absence of sub‐dividing or ‘double’ shoots were analyzed using a generalized linear mixed effects model (GLMM) with the same design but using family as binomial. The effects of the repeated clipping treatment on leaf production rates and tissue chemical traits were tested using linear mixed models with ‘treatment’ as a fixed factor and ‘plot’ as a random factor. The effects of repeated clipping treatment on the presence or absence of sub‐dividing or ‘double’ shoots were analyzed using a generalized linear mixed effects model (GLMM) with the same design as for productivity, but with family as binomial.

All linear mixed models were carried out using the R package ‘lme4’ (Bates et al. 2015), and all figures were produced using ‘ggplot2’ (Wickham 2016). Likelihood ratio tests between full and reduced models were used to carry out inference for the fixed effects using the ANOVA function. Tukey's HSD post hoc tests were used to test for between‐treatment differences. Residual plots were used to check all data for normality and homogeneity of variances, and data were transformed where appropriate (Appendix S1).

## 3 Results

### 3.1 Latitudinal Variation in Leaf Production and Responses to Simulated Herbivory

**FIGURE 2:**
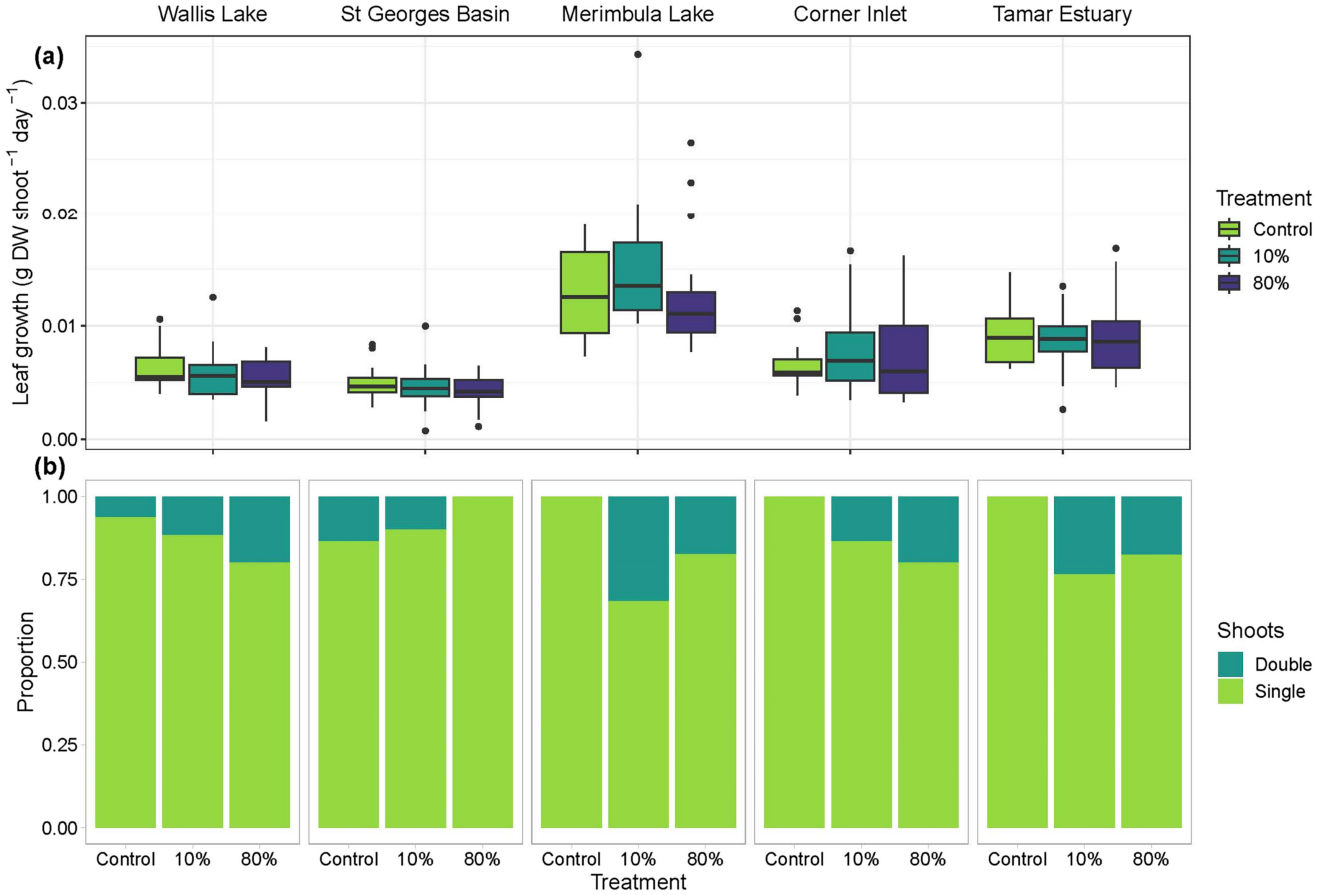
(a) Leaf growth (g DW shoot^−1^ day^−1^), and (b) proportion of shoots with additional growth modules (double shoots) of *P. australis* subjected to simulated herbivory treatments: Control, low (10%), and high (80%) intensity. Sites are ordered north to south by latitude, *n* = 21–30 shoots per treatment for growth. DW = dry weight. Box plots display the minimum, maximum, median, and quartiles.

Leaf production did not vary significantly with latitude and was also not affected by clipping (*p* = 0.37, *p* = 0.52, respectively; Figure 2a, Table S1a). Seagrasses subjected to simulated herbivory displayed similar leaf production rates as unclipped controls (Figure 2a, Table S1a), regardless of latitude and regardless of whether leaf growth was measured as new biomass (average rate across all treatments = 0.008 g DW shoot^−1^ day^−1^ ± 0.0002 SE, Figure 2a, Table S1a) or new area (average rate across all treatments = 1.55 cm^2^ shoot^−1^ day^−1^ ± 0.047, Figure S4b, Table S3b). Similar patterns were observed when production rate was measured per square meter (i.e., multiplying either new leaf biomass or new leaf area by the mean shoot density at each location; Figure S4a,c; Table S3a,c). However, the clipping treatment did have a significant effect on the architecture of *P. australis* shoots, with the presence of double shoots being overall higher in clipped plots regardless of latitude (*p* < 0.05; Figure 2b, Table S1d). Compared to controls, the presence of double shoots was 3.35 and 4.16 times higher in the 80% and 10% treatments, respectively. Tukey's post hoc comparisons confirmed that double shoots were higher in the 10% treatment plots compared to control plots (*p* = 0.03).

Low levels of natural herbivory on *P. australis* were observed in the region. Only 7% of removed shoots showed signs of natural herbivory, with leaf area removed by bites being only 0.1 ± 0.03 cm^2^ per shoot (less than 1% of the total leaf area).

**FIGURE 3:**
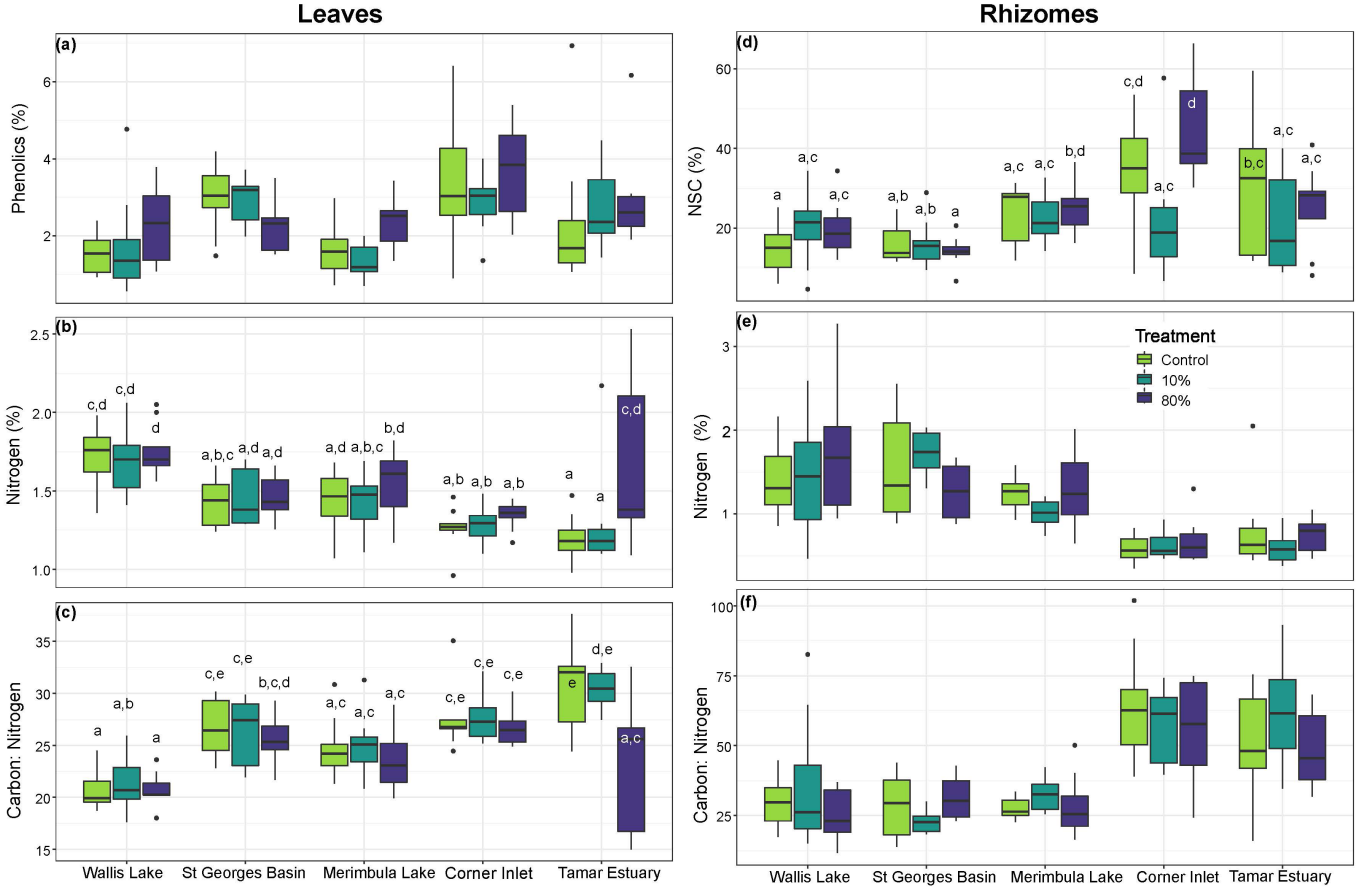
Tissue chemical traits of leaves (a–c), and rhizomes (d–f) of *P. australis* shoots subjected to simulated herbivory treatments: Control (C), low (10%), and high (80%) intensity. Specifically, (a) leaf phenolic content, (b) leaf nitrogen content, and (c) leaf C:N ratio, (d) rhizome total non‐structural carbohydrate (NSC) content, (e) rhizome nitrogen content and (c) rhizome C:N ratio. Sites are ordered north to south by latitude, *n* = 9 shoots per treatment for all chemical traits. DW = dry weight. Boxes labeled with the same lower‐case letter do not differ significantly according to the Tukey HSD test (*p* < 0.05). Box plots display the minimum, maximum, median, and quartiles.

There were no effects of latitude or the simulated herbivory treatment on phenolic content of the leaves (*p* = 0.18, *p* = 0.27, respectively; Figure 3a, Table S1c) or leaf carbon concentration (*p* = 0.58, *p* = 0.20, respectively; Table S1i). Overall, leaves had an average of 2.47% ± 0.10 SE in phenolics, 1.46% ± 0.02 SE in Nitrogen and 35.90% ± 0.12 SE in Carbon contents. There were significant effects for ‘treatment by latitude’, ‘treatment’ and ‘latitude’ for leaf nitrogen concentration (*p* < 0.05; Table S1h), which decreased with increasing latitude overall (Figure 3b). The lowest latitude site Wallis Lake displayed higher leaf nitrogen levels than the two highest latitude sites (Corner Inlet and Tamar Estuary), except for the 80% herbivory treatment at Tamar Estuary which was higher relative to the control and 10% treatments (Figure 3b). There were significant effects for ‘treatment by latitude’, ‘treatment’ and ‘latitude’ for C:N ratio in leaves (*p* < 0.05, *p* < 0.05, *p* < 0.01, respectively; Table S1j). Overall, the C:N ratio of the leaves increased with increasing latitude (leaves in Tamar Estuary had 1.4 times higher C:N ratio compared to those in Wallis Lake) and was significantly lower in the 80% treatment plots compared to control and 10% plots at the highest latitude site Tamar Estuary (Figure 3c).

Rhizome total non‐structural carbohydrates (NSC) ranged on average from 15.32% ± 0.81 SE in St Georges Basin to 33.13% ± 3.07 SE in Corner Inlet. The effects of clipping on total (NSC) of rhizomes varied with latitude (interaction, *p* < 0.05; Figure 3d, Table S1b). Overall rhizomes contained 1.15% ± 0.04 SE of Nitrogen and 35.49% ± 0.24 SE of Carbon. Rhizome nitrogen concentration (Figure 3e), rhizome carbon concentration and C:N ratio in the rhizomes (Figure 3f) varied only with latitude (*p* < 0.01, *p* < 0.05, *p* < 0.05, respectively; Table S1e–g). The two highest latitude sites, Corner Inlet and Tamar Estuary, had the lowest %N and highest C:N ratio in the rhizomes (C:N ratio of 59.1 ± 3.28 and 54.0 ± 3.86, respectively; Figure 3e,f).

### 3.2 Temporal Patterns in Leaf Production, Chemical Traits and Responses to Simulated Herbivory at the Warmest Range‐Edge Population

**FIGURE 4:**
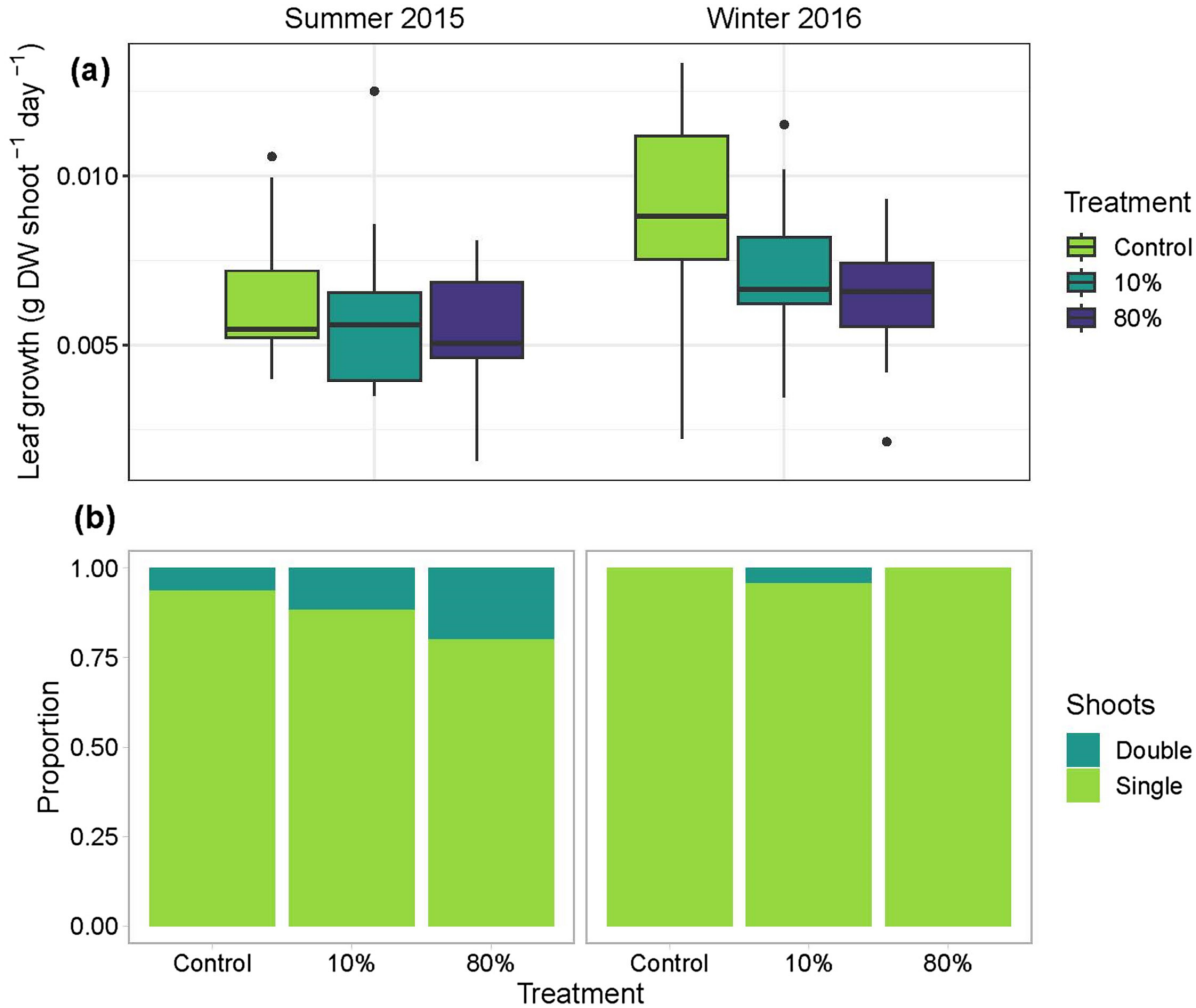
(a) Leaf growth (g DW shoot^−1^ day^−1^) and (b) proportion of shoots with additional growth modules (double shoots) of *P. australis* subjected to simulated herbivory treatments: Control, low (10%) and high (80%) intensity at the northernmost site, Wallis Lake, during austral summer 2015 and winter 2016. *n* = 21–30 shoots per treatment per sampling time for growth. DW = dry weight. Box plots display the minimum, maximum, median, and quartiles.

At the range‐edge meadow (Wallis Lake), leaf growth by biomass per shoot (g DW shoot^−1^ day^−1^) was 1.2 times higher in the winter than in the summer (*p* < 0.01; Figure 4a, Table S2a). The effect of treatment was also significant, albeit only marginally so (*p* = 0.047, Table S2a), and Tukey HSD post hoc tests could not resolve significant differences among pair‐wise comparisons. The architecture of the shoots was significantly affected by ‘time’ with 8.5 times more double shoots overall in the summer period compared to winter (*p* < 0.05; Figure 4b, Table S2d).

**FIGURE 5:**
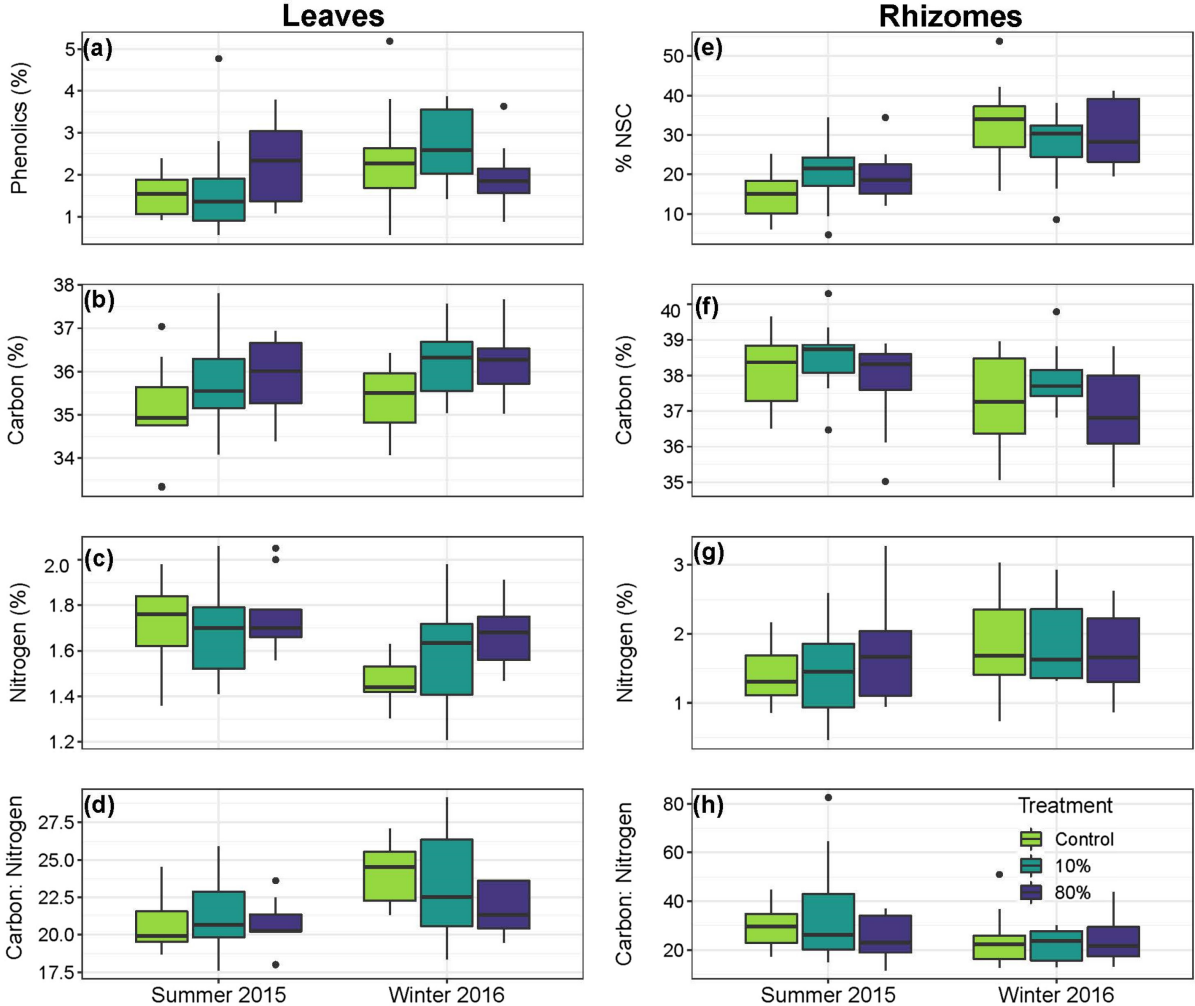
Tissue chemical traits of leaves (a–d) and rhizomes (e–h) of *P. australis* shoots subjected to simulated herbivory treatments: Control (C), low (10%), and high (80%) intensity at the northernmost site, Wallis Lake, during austral summer 2015 and winter 2016. Specifically, (a) leaf phenolic content, (b) leaf carbon content, (c) leaf nitrogen content, (d) leaf carbon: Nitrogen ratio, (e) rhizome total non‐structural carbohydrate (NSC) content, (f) rhizome carbon content, (g) rhizome nitrogen content, and (h) rhizome C:N ratio. *n* = 9 shoots per treatment for all chemical traits. Box plots display the minimum, maximum, median, and quartiles.

The clipping treatment resulted in higher leaf carbon content over both summer and winter sampling periods, albeit only marginally so (*p* = 0.049; Figure 5b, Table S2i). Tukey HSD post hoc tests could not resolve statistically significant differences among pair‐wise comparisons. None of the other chemical traits were affected by treatment, but some differed between summer and winter at Wallis Lake, with overall higher rhizome %NSC (*p* < 0.001; Figure 5e, Table S2b), and C:N ratio in the leaves (*p* < 0.01; Figure 5d, Table S2j) in winter, and lower leaf nitrogen (*p* < 0.05; Figure 5c, Table S2h) and rhizome carbon (*p* < 0.05; Figure 5f, Table S2f) in winter. Time or treatment did not influence leaf phenolics (*p* = 0.06, *p* = 0.77, respectively; Figure 5a, Table S2c), rhizome nitrogen content (*p* = 0.46, *p* = 0.99, respectively; Figure 5g, Table S2e) and rhizome C:N ratio (*p* = 0.29, *p* = 0.82, respectively; Figure 5h, Table S2g).

### 3.3 Responses to Repeated Simulated Herbivory at the Warmest Range‐Edge Population

The effect of clipping on leaf growth in Wallis Lake disappeared following repeated clipping treatments, with no differences among treatments on any of the production rates or on the formation of double shoots (Appendix S2, Figure S5). Repeatedly clipping the leaves did not have a significant effect on any of the chemical traits measured (Appendix S2, Figure S6).

## 4 Discussion

This study shows that *Posidonia australis* can tolerate high levels of herbivory, with losses of up to 80% of leaf biomass without any negative impacts on growth, and this is consistent along the full latitudinal range of the species. This result was upheld even after a period of repeated simulated herbivory at the warmest range‐edge seagrass population. The main effect of simulating herbivory on *Posidonia australis* along a latitudinal gradient was on the architecture of the seagrass, whereby clipped plants responded to damage by subdividing and producing new shoots. Thus, this important seagrass may be relatively resilient to future increases in herbivory driven by warming oceans.

There is a limited understanding of the ability of seagrasses to tolerate herbivory at different temperatures or latitudinal scales. The findings from this study are consistent with a small but growing body of literature that shows high resilience of temperate seagrasses to increases in herbivory. For example, Sanmartí et al. (2014) found leaf biomass in the species *Cymodocea nodosa* was similar in high herbivory and control plots after repeated clipping events, with plants producing additional modules (leaves and shoots) as a compensatory response to herbivory. The increase in production of subdividing shoots in clipped plots found in our study could potentially be a strategy of the plant to tolerate the effects of increased herbivory by producing more shoots to capitalize on increased light arriving to the basal parts of the plant. Similarly, other temperate seagrass species are able to maintain or even increase leaf regrowth rates following simulated grazing events (Vergés et al. 2008; Garthwin, Poore, and Vergés 2014), and the slow‐growing Mediterranean seagrass *Posidonia oceanica* is known to persist despite high consumption rates by fish (Tomas, Turon, and Romero 2005; Bennett et al. 2022). Direct mechanisms behind this resistance include the presence of extensive underground rhizome mats and the ability to translocate nutrients from the rhizome (Alcoverro, Manzanera, and Romero 2001; Marbà, Hemminga, and Duarte 2006), however, indirect mechanisms (e.g., the modification of a plant trait that in turn reduces its consumption) are also emerging as critical (Buñuel et al. 2023).

In contrast, the response of tropical seagrasses to herbivory is more variable, with some able to maintain or increase growth following herbivory (Kuiper‐Linley, Johnson, and Lanyon 2007; Gulick et al. 2020), while others respond to increased herbivory with a rapid decline in seagrass biomass (Rodriguez and Heck 2020) and productivity (Campbell et al. 2024). In general, faster growing tropical seagrasses sustain higher levels of herbivory than temperate, slow growing species like *P. australis* (Nowicki, Fourqurean, and Heithaus 2018), however increased grazing intensities can lead to longer recovery time of the colonizing seagrass *Halophila ovalis* in a temperate estuary (O'Dea et al. 2022). The meadows studied here were characterized by very low levels of natural herbivory, as less than 1% of leaf area was consumed. This is in contrast to other temperate species, where up to 30% of primary production may be consumed (Vergés et al. 2018). In any case, the ability of *P. australis* to recover from high levels of cropping suggests that this species may have co‐evolved with high levels of herbivory in the past (Heck Jr and Valentine 2006).

Some seagrasses are able to adapt to losses in biomass by changing plant traits, however the patterns of maintained growth observed in this study could not be explained by any of the plant traits analyzed. Our findings contrast to other simulated herbivory experiments in other seagrasses where tolerance to biomass losses correlated with observed changes to plant traits (Vergés et al. 2008; Sanmartí et al. 2014). For example, reductions in carbohydrate reserves in the rhizomes of *Cymodocea* spp. occurred in response to simulated herbivory (Kuiper‐Linley, Johnson, and Lanyon 2007; Sanmartí et al. 2014). It was surprising that nonstructural carbohydrates were not depleted in rhizomes of clipped shoots in this study, including after repeated clipping events at the northernmost site, given the expectation that plots subjected to 80% loss would reallocate resources from the rhizome to maintain leaf growth rates. One potential explanation is that leaf growth was maintained using nonstructural carbohydrates stores in the leaves rather than rhizomes (Kuiper‐Linley, Johnson, and Lanyon 2007). Seagrass shoots are known to translocate resources (including photosynthates) between shoots of the same rhizome (Marbà, Hemminga, and Duarte 2006; Prado, Alcoverro, and Romero 2008). However, in this study, translocation of reserves from unclipped to clipped shoots is unlikely to have occurred in 1.5 m × 1.5 m clipped plots, as maximal distances for nutrient translocation within rhizomes of *Posidonia oceanica* in the Mediterranean is less than 20 cm (Marbà et al. 2002). Novel molecular techniques, such as metabolomics to investigate seagrass metabolic pathways, could contribute to unravel plant responses and identify early indicators of plant stress (Jung et al. 2022, 2023).

*Posidonia* spp. generally have higher biomass growth in summer, when light availability is the highest, which is also when higher herbivory pressure has been observed (Alcoverro, Duarte, and Romero 1995; Cambridge and Hocking 1997; Tomas, Turon, and Romero 2005). Despite the variability in responses to the simulated herbivory treatment, the small but significant negative effect of clipping on seagrass growth during winter suggests that seagrasses may be more vulnerable to herbivory in winter, when shorter daylengths may be limiting photosynthesis (noting that there are fewer studies investigating growth during winter; Heck et al. 2021). Surprisingly, however, leaf elongation rates were higher in winter than in the summer. This is the opposite trend to West and Larkum (1979), Dennison (1987) and Cambridge and Hocking (1997). This could be explained by the different strategy observed in this study whereby plants produced more double shoots in summer compared to the winter sampling period.

There was a trend toward higher total nonstructural carbohydrates in the winter compared to the summer sampling period at the warmest range‐edge site Wallis Lake. This was unexpected, as these nutrient reserves are usually used to sustain the plants during the period of decreased productivity in winter, due to plants utilizing starch stores for maintenance and growth (Dawes and Lawrence 1980), and rhizome carbohydrate stores of other seagrass species are at the highest in late spring or summer (Alcoverro, Manzanera, and Romero 2001; Collier et al. 2008). However, the current study only sampled one annual cycle, thus seasonal trends year to year cannot be determined, in addition, short‐term stress and small‐scale disturbance events may cause variations in carbon reserves (Soissons et al. 2018). Interestingly, in winter, carbohydrate depletion was not observed in clipped plots compared to control plots. It was expected that the plants would rely more heavily on carbohydrate reserves during the colder months to maintain growth rates after being clipped. However, this is consistent with Vergés et al. (2008) who found no impact of simulated herbivory on *Posidonia oceanica* rhizome carbohydrate stores. Overall, given the variability of seagrass responses to herbivory, investigating the sugars metabolic pathway could help clarify those trends in carbohydrates.

The results of this study contrast with findings from other studies that have examined latitudinal patterns in seagrass growth and plant traits. Firstly, this study found no latitudinal trends in growth rates of *Posidonia australis*. In contrast, Vergés et al. (2018) found strong latitudinal patterns in growth rates of *Amphibolis antarctica* on the west coast of Australia, with higher growth in the tropics than in temperate latitudes. Secondly, content of nitrogen in *P. australis* decreased with latitude, whereas global studies of terrestrial and aquatic plants generally find that leaf nitrogen content increases in higher latitudes (Reich and Oleksyn 2004; Vergés et al. 2018). However, there is no clear pattern about the relationship between nitrogen and herbivore feeding patterns in seagrass‐dominated ecosystems (Valentine and Heck 2021). The observed latitudinal patterns in both growth and leaf nitrogen may be related to site‐specific characteristics of the locations where *P. australis* occurs along the eastern coast of Australia, which differ along the latitudinal coastline. The two highest latitude sites (Corner Inlet and Tamar Estuary) are more oceanic than the other three sites, which are semi‐enclosed estuaries or lakes (Figure 1). This could mean that factors other than latitude or temperature (which varies predictably with latitude) were responsible for the observed patterns (Heck et al. 2021). There is evidence that defense strategies against herbivory (e.g., growth and tissue nutrients) are highly influenced by other factors, such as nutrient availability, thus may not respond linearly to latitude or increased herbivory (Hernán et al. 2020). Estuarine seagrass habitats can be characterized by higher nutrient levels due to riverine inputs, less flushing, and high variability due to fluctuating temperature, light and salinity (Carruthers et al. 2007), potentially explaining the lower leaf nitrogen content in the two more oceanic sites.

This study shows that *Posidonia australis* has a remarkable resilience to damage caused by simulated herbivory, and this species was able to maintain similar growth rates at all latitudes despite losing up to 80% of leaf biomass. Thus, this important and regionally‐endangered seagrass may be more resilient to future increases in herbivory than other habitats like kelp, which are already declining in response to increases in herbivory (Bennett et al. 2015; Vergés et al. 2016). It is important to note that, while for seaweeds Toth and Pavia (2007) did not find a difference in induced responses between artificial and natural damage, to date there is no such evidence for seagrasses. Further investigation comparing plants damaged by different levels of natural fish herbivory to mechanical simulations of herbivory and undamaged plants is needed to clarify whether the observed responses match real herbivory events.

## 5 Conclusions

In a changing climate and associated alterations in species interactions, there is mounting evidence that there will be ‘winners and losers’. Evidence to date shows that although seagrasses are certainly being impacted by ocean warming and heatwave events (Strydom et al. 2020; Nguyen et al. 2021), they are comparatively more resilient than seaweeds. While the distribution of kelp and fucoid seaweeds has contracted in response to heatwaves more than 100 km in regions such as Western Australia (Wernberg et al. 2016), seagrasses in the same regions are still present, albeit at reduced densities, in impacted sites (Strydom et al. 2020). Our findings suggest that seagrasses such as *Posidonia australis* are relatively resilient to large variations in herbivory and may potentially end up being ‘winners’ under future climate change conditions as oceans continue to warm and consumer pressure from herbivorous fish increases.

## Author Contributions

**Ruby G. Garthwin:** conceptualization (equal), formal analysis (equal), investigation (equal), methodology (equal), visualization (equal), writing – original draft (lead), writing – review and editing (equal). **Alistair G. B. Poore:** conceptualization (equal), formal analysis (equal), methodology (equal), supervision (equal), writing – review and editing (equal). **Giulia Ferretto:** formal analysis (equal), visualization (equal), writing – review and editing (equal). **Jeffrey T. Wright:** conceptualization (supporting), methodology (equal), writing – review and editing (equal). **Adriana Vergés:** conceptualization (equal), funding acquisition (lead), investigation (equal), methodology (equal), supervision (equal), writing – review and editing (equal).

## Conflicts of Interest

The authors declare no conflicts of interest.

## Supporting information


Data S1.

## Data Availability

Data and codes can be found here: https://doi.org/10.5281/zenodo.10566147.

## References

[ece370561-bib-0001] Agrawal, A. A. , and J. L. Maron . 2022. “Long‐Term Impacts of Insect Herbivores on Plant Populations and Communities.” Journal of Ecology 110: 2800–2811.

[ece370561-bib-0002] Alcoverro, T. , C. M. Duarte , and J. Romero . 1995. “Annual Growth Dynamics of *Posidonia oceanica* : Contribution of Large‐Scale Versus Local Factors to Seasonality.” Marine Ecology Progress Series 120: 203–210.

[ece370561-bib-0003] Alcoverro, T. , M. Manzanera , and J. Romero . 2001. “Annual Metabolic Carbon Balance of the Seagrass *Posidonia oceanica* : The Importance of Carbohydrate Reserves.” Marine Ecology Progress Series 211: 105–116.

[ece370561-bib-0004] Bates, D. , M. Mächler , B. Bolker , and S. Walker . 2015. “Fitting Linear Mixed‐Effects Models Using lme4.” Journal of Statistical Software 67: 1–48.

[ece370561-bib-0201] Bell, J. D. , J. J. Burchmore , and D. A. Pollard . 1978. “Feeding ecology of three sympatric species of leatherjackets (Pisces: Monacanthidae) from a Posidonia seagrass habitat in New South Wales.” Marine and Freshwater Research 29: 631–643.

[ece370561-bib-0005] Bennett, S. , T. Alcoverro , D. Kletou , et al. 2022. “Resilience of Seagrass Populations to Thermal Stress Does Not Reflect Regional Differences in Ocean Climate.” New Phytologist 233: 1657–1666. 34843111 10.1111/nph.17885 PMC9299911

[ece370561-bib-0006] Bennett, S. , T. Wernberg , E. S. Harvey , J. Santana‐Garcon , and B. J. Saunders . 2015. “Tropical Herbivores Provide Resilience to a Climate‐Mediated Phase Shift on Temperate Reefs.” Ecology Letters 18: 714–723. 25994785 10.1111/ele.12450

[ece370561-bib-0007] Blois, J. L. , P. L. Zarnetske , M. C. Fitzpatrick , and S. Finnegan . 2013. “Climate Change and the Past, Present, and Future of Biotic Interactions.” Science 341: 499–504. 23908227 10.1126/science.1237184

[ece370561-bib-0008] Bolser, R. C. , M. E. Hay , N. Lindquist , W. Fenical , and D. Wilson . 1998. “Chemical Defenses of Freshwater Macrophytes Against Crayfish Herbivory.” Journal of Chemical Ecology 24: 1639–1658.

[ece370561-bib-0009] Buñuel, X. , T. Alcoverro , J. Boada , et al. 2023. “Indirect Grazing‐Induced Mechanisms Contribute to the Resilience of Mediterranean Seagrass Meadows to sea Urchin Herbivory.” Oikos 2023: e09520.

[ece370561-bib-0010] Buñuel, X. , T. Alcoverro , J. Romero , et al. 2021. “Warming Intensifies the Interaction Between the Temperate Seagrass *Posidonia oceanica* and Its Dominant Fish Herbivore *Sarpa salpa* .” Marine Environmental Research 165: 105237. 33476979 10.1016/j.marenvres.2020.105237

[ece370561-bib-0011] Burkepile, D. E. , and J. D. Parker . 2017. “Recent Advances in Plant‐Herbivore Interactions.” F1000Research 6: 119. 28232868 10.12688/f1000research.10313.1 PMC5302155

[ece370561-bib-0012] Cambridge, M. L. , and P. J. Hocking . 1997. “Annual Primary Production and Nutrient Dynamics of the Seagrasses *Posidonia sinuosa* and *Posidonia australis* in South‐Western Australia.” Aquatic Botany 59: 277–295.

[ece370561-bib-0013] Campbell, J. E. , R. O. Kennedy , C. J. Munson , et al. 2024. “Herbivore Effects Increase With Latitude Across the Extent of a Foundational Seagrass.” Nature Ecology and Evolution 16: 1–13. 10.1038/s41559-024-02336-5 38366132

[ece370561-bib-0014] Carruthers, T. , W. Dennison , G. Kendrick , M. Waycott , D. Walker , and M. Cambridge . 2007. “Seagrasses of South–West Australia: A Conceptual Synthesis of the World's Most Diverse and Extensive Seagrass Meadows.” Journal of Experimental Marine Biology and Ecology 350: 21–45.

[ece370561-bib-0015] Collier, C. J. , P. S. Lavery , P. J. Ralph , and R. J. Masini . 2008. “Physiological Characteristics of the Seagrass *Posidonia Sinuosa* Along a Depth‐Related Gradient of Light Availability.” Marine Ecology Progress Series 353: 65–79.

[ece370561-bib-0016] Corlett, R. T. , and D. A. Westcott . 2013. “Will Plant Movements Keep Up With Climate Change?” Trends in Ecology & Evolution 28: 482–488. 23721732 10.1016/j.tree.2013.04.003

[ece370561-bib-0017] Dawes, C. J. , and J. M. Lawrence . 1980. “Seasonal Changes in the Proximate Constituents of the Seagrasses *Thalassia testudinum* , *Halodule wrightii* . and *Syringodium filiforme* .” Aquatic Botany 8: 371–380.

[ece370561-bib-0018] De los Santos, C. B. , A. Scott , A. Arias‐Ortiz , et al. 2020. “Seagrass Ecosystem Services: Assessment and Scale of Benefits.” In Out of the Blue: The Value of Seagrasses to the Environment and to People, edited by M. Potouroglou , 19–34. Nairobi: United Nations Environment Programme (UNEP).

[ece370561-bib-0019] Dennison, W. C. 1987. “Effects of Light on Seagrass Photosynthesis, Growth and Depth Distribution.” Aquatic Botany 27: 15–26.

[ece370561-bib-0020] Duarte, C. M. , and D. Krause‐Jensen . 2017. “Export From Seagrass Meadows Contributes to Marine Carbon Sequestration.” Frontiers in Marine Science 4: 13.

[ece370561-bib-0021] Evans, S. M. , K. J. Griffin , R. A. J. Blick , A. G. B. Poore , and A. Vergés . 2018. “Seagrass on the Brink: Decline of Threatened Seagrass *Posidonia australis* Continues Following Protection.” PLoS One 13: 1–18. 10.1371/journal.pone.0190370 PMC5889071 29624579

[ece370561-bib-0022] Garthwin, R. G. , A. G. Poore , and A. Vergés . 2014. “Seagrass Tolerance to Herbivory Under Increased Ocean Temperatures.” Marine Pollution Bulletin 83: 475–482. 23993389 10.1016/j.marpolbul.2013.08.010

[ece370561-bib-0023] Gobert, S. , M. T. Cambridge , B. Velimirov , et al. 2006. “Biology of *Posidonia* .” In Seagrasses: Biology, Ecology and Conservation, edited by A. W. D. Larkum , R. J. Orth , and C. M. Duarte , 387–408. Dordrecht, The Netherlands: Springer.

[ece370561-bib-0024] Gulick, A. G. , R. A. Johnson , C. G. Pollock , Z. Hillis‐Starr , A. B. Bolten , and K. A. Bjorndal . 2020. “Recovery of a Large Herbivore Changes Regulation of Seagrass Productivity in a Naturally Grazed Caribbean Ecosystem.” Ecology 101: e03180. 32882749 10.1002/ecy.3180

[ece370561-bib-0025] Hamann, E. , C. Blevins , S. J. Franks , M. I. Jameel , and J. T. Anderson . 2020. “Climate Change Alters Plant‐Herbivore Interactions.” New Phytologist 229: 1894–1910. 33111316 10.1111/nph.17036

[ece370561-bib-0026] Heck, K. L., Jr. , and J. F. Valentine . 2006. “Plant–Herbivore Interactions in Seagrass Meadows.” Journal of Experimental Marine Biology and Ecology 330: 420–436.

[ece370561-bib-0027] Heck, K. L. , M. Samsonova , A. G. B. Poore , and G. A. Hyndes . 2021. “Global Patterns in Seagrass Herbivory: Why, Despite Existing Evidence, There Are Solid Arguments in Favor of Latitudinal Gradients in Seagrass Herbivory.” Estuaries and Coasts 44: 481–490.

[ece370561-bib-0028] Hernán, G. , M. J. Ortega , A. M. Gandara , I. Castejon , J. Terrados , and F. Tomas . 2017. “Future Warmer Seas: Increased Stress and Susceptibility to Grazing in Seedlings of a Marine Habitat‐Forming Species.” Global Change Biology 23: 4530–4543. 28544549 10.1111/gcb.13768

[ece370561-bib-0029] Hernán, G. , M. J. Ortega , J. Henderson , et al. 2020. “Latitudinal Variation in Plant Defence Against Herbivory in a Marine Foundation Species Does Not Follow a Linear Pattern: The Importance of Resource Availability.” Global Ecology and Biogeography 30: 220–234.

[ece370561-bib-0030] Hyndes, G. A. , K. L. Heck Jr. , A. Vergés , et al. 2016. “Accelerating Tropicalization and the Transformation of Temperate Seagrass Meadows.” Bioscience 66: 938–948. 28533562 10.1093/biosci/biw111 PMC5421442

[ece370561-bib-0031] Jiménez‐Ramos, R. , L. G. Egea , M. J. Ortega , I. Hernández , J. J. Vergara , and F. G. Brun . 2017. “Global and Local Disturbances Interact to Modify Seagrass Palatability.” PLoS One 12: e0183256. 28813506 10.1371/journal.pone.0183256 PMC5558941

[ece370561-bib-0032] Jung, E. M. U. , J. J. Cosgrove , B. C. Martin , M. Bollen , G. A. Kendrick , and M. W. Fraser . 2022. “Seasonal Links Between Metabolites and Traditional Seagrass Metrics in the Seagrass in an Estuarine System.” Ecological Indicators 143: 109315.

[ece370561-bib-0033] Jung, E. M. U. , N. A. B. A. Majeed , M. W. Booth , et al. 2023. “Marine Heatwave and Reduced Light Scenarios Cause Species‐Specific Metabolomic Changes in Seagrasses Under Ocean Warming.” New Phytologist 239: 1692–1706. 37357353 10.1111/nph.19092

[ece370561-bib-0034] Kuiper‐Linley, M. , C. R. Johnson , and J. M. Lanyon . 2007. “Effects of Simulated Green Turtle Regrazing on Seagrass Abundance, Growth and Nutritional Status in Moreton Bay, South‐East Queensland, Australia.” Marine and Freshwater Research 58: 492–503.

[ece370561-bib-0035] Kumagai, N. H. , J. G. Molinos , H. Yamano , S. Takao , M. Fujii , and Y. Yamanaka . 2018. “Ocean Currents and Herbivory Drive Macroalgae‐To‐Coral Community Shift Under Climate Warming.” Proceedings of the National Academy of Sciences of the United States of America 115: 8990–8995. 30126981 10.1073/pnas.1716826115 PMC6130349

[ece370561-bib-0036] Lenoir, J. , R. Bertrand , L. Comte , et al. 2020. “Species Better Track Climate Warming in the Oceans Than on Land.” Nature Ecology & Evolution 4: 1044–1059. 32451428 10.1038/s41559-020-1198-2

[ece370561-bib-0037] Longo, G. O. , M. E. Hay , C. E. L. Ferreira , and S. R. Floeter . 2018. “Trophic Interactions Across 61 Degrees of Latitude in the Western Atlantic.” Global Ecology and Biogeography 28: 107–117.

[ece370561-bib-0038] Marbà, N. , M. A. Hemminga , and C. M. Duarte . 2006. “Resource Translocation Within Seagrass Clones: Allometric Scaling to Plant Size and Productivity.” Oecologia 150: 362–372. 16944245 10.1007/s00442-006-0524-y

[ece370561-bib-0039] Marbà, N. , M. A. Hemminga , M. A. Mateo , et al. 2002. “Carbon and Nitrogen Translocation Between Seagrass Ramets.” Marine Ecology Progress Series 226: 287–300.

[ece370561-bib-0040] Mcnaughton, S. J. , M. Oesterheld , D. A. Frank , and K. J. Williams . 1989. “Ecosystem‐Level Patterns of Primary Productivity and Herbivory in Terrestrial Habitats.” Nature 341: 142–144. 2779651 10.1038/341142a0

[ece370561-bib-0041] Nguyen, H. M. , P. J. Ralph , L. Marin‐Guirao , M. Pernice , and G. Procaccini . 2021. “Seagrasses in an Era of Ocean Warming: A Review.” Biological Reviews 96: 2009–2030. 34014018 10.1111/brv.12736

[ece370561-bib-0042] Nowicki, R. J. , J. W. Fourqurean , and M. R. Heithaus . 2018. “The Role of Consumers in Structuring Seagrass Communities: Direct and Indirect Mechanisms: Structure, Ecology and Conservation.” In Seagrasses of Australia, edited by A. W. D. Larkum , G. A. Kendrick , and P. Ralph , 491–540. Cham: Springer.

[ece370561-bib-0043] Ockendon, N. , D. J. Baker , J. A. Carr , et al. 2014. “Mechanisms Underpinning Climatic Impacts on Natural Populations: Altered Species Interactions Are More Important Than Direct Effects.” Global Change Biology 20: 2221–2229. 24677405 10.1111/gcb.12559

[ece370561-bib-0044] O'Dea, C. M. , P. S. Lavery , C. L. Webster , and K. M. McMahon . 2022. “Increased Extent of Waterfowl Grazing Lengthens the Recovery Time of a Colonizing Seagrass ( *Halophila ovalis* ) With Implications for Seagrass Resilience.” Frontiers in Plant Science 13: 947109. 36105704 10.3389/fpls.2022.947109 PMC9465301

[ece370561-bib-0045] Pagès, J. F. , T. M. Smith , F. Tomas , et al. 2018. “Contrasting Effects of Ocean Warming on Different Components of Plant‐Herbivore Interactions.” Marine Pollution Bulletin 134: 55–65. 29074253 10.1016/j.marpolbul.2017.10.036

[ece370561-bib-0046] Poore, A. G. B. , A. H. Campbell , R. A. Coleman , et al. 2012. “Global Patterns in the Impact of Marine Herbivores on Benthic Primary Producers.” Ecology Letters 15: 912–922. 22639820 10.1111/j.1461-0248.2012.01804.x

[ece370561-bib-0047] Prado, P. , T. Alcoverro , and J. Romero . 2008. “Seasonal Response of *Posidonia oceanica* Epiphyte Assemblages to Nutrient Increase.” Marine Ecology Progress Series 359: 89–98.

[ece370561-bib-0048] Reich, P. B. , and J. Oleksyn . 2004. “Global Patterns of Plant Leaf N and P in Relation to Temperature and Latitude.” Proceedings of the National Academy of Sciences of the United States of America 101: 11001–11006. 15213326 10.1073/pnas.0403588101 PMC503733

[ece370561-bib-0049] Rodriguez, A. R. , and K. L. Heck . 2020. “Green Turtle Herbivory and Its Effects on the Warm, Temperate Seagrass Meadows of St. Joseph Bay, Florida (USA).” Marine Ecology Progress Series 639: 37–51.

[ece370561-bib-0050] Romero, J. 1989. “Primary Production of *Posidonia oceanica* Beds in the Medas Islands (Girona, NE Spain).” In Proceeding of the International Workshop on Posidonia oceanica Beds, edited by C. F. Boudouresque , A. Meinesz , E. Fresi , and V. Gravez , 83–86. Marseille, France: GIS Posidonie.

[ece370561-bib-0051] Roslin, T. , B. Hardwick , V. Novotny , et al. 2017. “Higher Predation Risk for Insect Prey at Low Latitudes and Elevations.” Science 356: 742–744. 28522532 10.1126/science.aaj1631

[ece370561-bib-0052] Samsonova, M. 2020. Tropicalisation of Temperate Seagrass Meadows in Western Australia: Predicting the Impact of Tropical Herbivorous Fishes on Temperate Seagrass Meadows. Perth: Edith Cowan University. https://ro.ecu.edu.au/theses/2294.

[ece370561-bib-0053] Sanmartí, N. , L. Saiz , I. Llagostera , M. Perez , and J. Romero . 2014. “Tolerance Responses to Simulated Herbivory in the Seagrass *Cymodocea nodosa* .” Marine Ecology Progress Series 517: 159–169.

[ece370561-bib-0054] Santana‐Garcon, J. , S. Bennett , N. Marbà , A. Vergés , R. Arthur , and T. Alcoverro . 2023. “Tropicalization Shifts Herbivore Pressure From Seagrass to Rocky Reef Communities.” Proceedings of the Royal Society B: Biological Sciences 290: 20221744. 10.1098/rspb.2022.1744 PMC9832549 36629100

[ece370561-bib-0055] Schleuning, M. , E. L. Neuschulz , J. Albrecht , et al. 2020. “Trait‐Based Assessments of Climate‐Change Impacts on Interacting Species.” Trends in Ecology & Evolution 35: 319–328. 31987640 10.1016/j.tree.2019.12.010

[ece370561-bib-0056] Scott, A. L. , P. H. York , and M. A. Rasheed . 2021. “Herbivory Has a Major Influence on Structure and Condition of a Great Barrier Reef Subtropical Seagrass Meadow.” Estuaries and Coasts 44: 506–521.

[ece370561-bib-0057] Soissons, L. M. , E. P. Haanstra , M. M. van Katwijk , et al. 2018. “Latitudinal Patterns in European Seagrass Carbon Reserves: Influence of Seasonal Fluctuations versus Short‐Term Stress and Disturbance Events.” Frontiers in Plant Science 9: 88. 29449859 10.3389/fpls.2018.00088 PMC5799261

[ece370561-bib-0058] Strydom, S. , K. Murray , S. Wilson , et al. 2020. “Too Hot to Handle: Unprecedented Seagrass Death Driven by Marine Heatwave in a World Heritage Area.” Global Change Biology 26: 3525–3538. 32129909 10.1111/gcb.15065

[ece370561-bib-0059] Tomas, F. , B. Martinez‐Crego , G. Hernán , and R. Santos . 2015. “Responses of Seagrass to Anthropogenic and Natural Disturbances Do Not Equally Translate to Its Consumers.” Global Change Biology 21: 4021–4030. 26152761 10.1111/gcb.13024

[ece370561-bib-0060] Tomas, F. , X. Turon , and J. Romero . 2005. “Seasonal and Small‐Scale Spatial Variability of Herbivory Pressure on the Temperate Seagrass *Posidonia oceanica* .” Marine Ecology Progress Series 301: 95–107.

[ece370561-bib-0061] Toth, G. B. , and H. Pavia . 2007. “Induced Herbivore Resistance in Seaweeds: A Meta‐Analysis.” Journal of Ecology 95: 425–434.

[ece370561-bib-0062] Unsworth, R. K. , J. D. Taylor , A. Powell , J. J. Bell , and D. J. Smith . 2007. “The Contribution of Scarid Herbivory to Seagrass Ecosystem Dynamics in the Indo‐Pacific.” Estuarine, Coastal and Shelf Science 74: 53–62.

[ece370561-bib-0063] Valentine, J. F. , and K. L. Heck . 2021. “Herbivory in Seagrass Meadows: An Evolving Paradigm.” Estuaries and Coasts 44: 491–505.

[ece370561-bib-0064] Vergés, A. , M. A. Becerro , T. Alcoverro , and J. Romero . 2007. “Variation in Multiple Traits of Vegetative and Reproductive Seagrass Tissues Influences Plant–Herbivore Interactions.” Oecologia 151: 675–686. 17120055 10.1007/s00442-006-0606-x

[ece370561-bib-0065] Vergés, A. , C. Doropoulos , R. Czarnik , K. McMahon , N. Llonch , and A. G. B. Poore . 2018. “Latitudinal Variation in Seagrass Herbivory: Global Patterns and Explanatory Mechanisms.” Global Ecology and Biogeography 27: 1068–1079.

[ece370561-bib-0066] Vergés, A. , C. Doropoulos , H. A. Malcolm , et al. 2016. “Long‐Term Empirical Evidence of Ocean Warming Leading to Tropicalization of Fish Communities, Increased Herbivory, and Loss of Kelp.” Proceedings of the National Academy of Sciences of the United States of America 113: 13791–13796. 27849585 10.1073/pnas.1610725113 PMC5137712

[ece370561-bib-0067] Vergés, A. , E. McCosker , M. Mayer‐Pinto , et al. 2019. “Tropicalisation of Temperate Reefs: Implications for Ecosystem Functions and Management Actions.” Functional Ecology 33: 1000–1013.

[ece370561-bib-0068] Vergés, A. , M. Pérez , T. Alcoverro , and J. Romero . 2008. “Compensation and Resistance to Herbivory in Seagrasses: Induced Responses to Simulated Consumption by Fish.” Oecologia 155: 751–760. 18193292 10.1007/s00442-007-0943-4

[ece370561-bib-0069] Vergés, A. , P. D. Steinberg , M. E. Hay , et al. 2014a. “The Tropicalization of Temperate Marine Ecosystems: Climate‐Mediated Changes in Herbivory and Community Phase Shifts.” Proceedings of the Royal Society B: Biological Sciences 281: 20140846. 10.1098/rspb.2014.0846 PMC4100510 25009065

[ece370561-bib-0070] Vergés, A. , F. Tomas , E. Cebrian , et al. 2014b. “Tropical Rabbitfish and the Deforestation of a Warming Temperate Sea.” Journal of Ecology 102: 1518–1527.

[ece370561-bib-0071] Wernberg, T. , S. Bennett , R. C. Babcock , et al. 2016. “Climate‐Driven Regime Shift of a Temperate Marine Ecosystem.” Science 353: 169–172. 27387951 10.1126/science.aad8745

[ece370561-bib-0072] West, G. J. , and T. M. Glasby . 2022. “Interpreting Long‐Term Patterns of Seagrasses Abundance: How Seagrass Variability Is Dependent on Genus and Estuary Type.” Estuaries and Coasts 45: 1393–1408.

[ece370561-bib-0073] West, R. , and A. Larkum . 1979. “Leaf Productivity of the Seagrass, *Posidonia australis*, in Eastern Australian Waters.” Aquatic Botany 7: 57–65.

[ece370561-bib-0074] Wickham, H. 2016. ggplot2: Elegant Graphics for Data Analysis. Cham: Springer.

[ece370561-bib-0075] Wressnig, A. , and D. J. Booth . 2008. “Patterns of Seagrass Biomass Removal by Two Temperate Australian Fishes (*Monacanthidae*).” Marine and Freshwater Research 59: 408–417.

[ece370561-bib-0076] Zarco‐Perello, S. , G. Carroll , M. Vanderklift , T. Holmes , T. J. Langlois , and T. Wernberg . 2020. “Range‐Extending Tropical Herbivores Increase Diversity, Intensity and Extent of Herbivory Functions in Temperate Marine Ecosystems.” Functional Ecology 34: 2411–2421.

[ece370561-bib-0077] Zvereva, E. L. , and M. V. Kozlov . 2021. “Latitudinal Gradient in the Intensity of Biotic Interactions in Terrestrial Ecosystems: Sources of Variation and Differences From the Diversity Gradient Revealed by Meta‐Analysis.” Ecology Letters 24: 2506–2520. 34322961 10.1111/ele.13851

