# Supplementary material for: Seagrass Tolerance to Simulated Herbivory Along a Latitudinal Gradient: Predicting the Potential Effects of Tropicalisation
Source: Ecol Evol. 2024 Nov 17;14(11):e70561. doi: 10.1002/ece3.70561 (PMC11570194; doi:10.1002/ece3.70561)
Supplement: Supplementary file 1 — Data S1. [file ECE3-14-e70561-s001.docx]

**Supporting Information**

*Appendix S1: transformations*

Log transformations were included for Wallis Lake repeated clipping chemical tissue analysis and sqrt transformations were applied to growth weight and shoot density data.

*Appendix S2: Responses to repeated simulated herbivory at the warmest range-edge seagrass population*

The effect of clipping on leaf growth in Wallis Lake disappeared following repeated clipping treatments, with no differences among treatments being observed regardless of whether testing for differences in new leaf growth by area (cm^2^shoot^-1^day^-1^) (*χ***^2^** = 2.72, df = 2, *p* = 0.26, Fig. S5a) or by biomass (g DW shoot ^-1^ day^-1^) (*χ* **^2^** = 3.47, df = 2, *p* = 0.18, Fig. S5b). Similarly, repeated clipping had no effect on the formation of double shoots (*χ* **^2^** = 1.37, df = 2, *p* = 0.51, Fig. S5c).

Repeatedly clipping the leaves did not have a significant effect on any of the chemical traits measured (Fig. S6): leaf phenolics (*χ* **^2^** = 2.50, df = 2, *p* = 0.29), % non-structural carbohydrates (*χ* **^2^** = 1.93, df = 2, *p* = 0.38), rhizome nitrogen (*χ* **^2^** = 1.12, df = 2, *p* = 0.57), rhizome carbon (*χ* **^2^** = 3.09, df = 2, *p* = 0.23), C:N ratio in the rhizomes (*χ* **^2^** = 0.5, df = 2, *p* = 0.78), leaf nitrogen (*χ* **^2^** = 2.6, df = 2, *p* = 0.88), leaf carbon (*χ* **^2^** = 2.2, df = 2, *p* = 0.33) or C:N ratio in the leaves (*χ* **^2^** = 0.54, df = 2, *p* = 0.76).

**Table S1.** Results of linear mixed models testing for differences between treatments and latitude on different traits of *Posidonia australis* shoots subjected to simulated herbivory treatments: control, low (10%) and high (80%) levels. Location and Plot were included in the model as random factors. Text in bold indicates significant (*p* < 0.05) differences. NSC = Total non-structural carbohydrates.

|  |  | |  |  |  |  |  |  |  |  |  |  |  |  |  |
| --- | --- | --- | --- | --- | --- | --- | --- | --- | --- | --- | --- | --- | --- | --- | --- |
|  | **(a) Leaf growth (g DW shoot ^-1^ day^-1^)** | | | | **(b) NSC** **rhizomes** | |  | **(c) Phenolics leaves** | | | **(d) Double shoots** | |  | **(e) Nitrogen rhizomes** | |
| **Source of variation** | *df* | *χ*^2^ | P |  | *χ*^2^ | P |  | *χ*^2^ | P |  | *χ*^2^ | P |  | *χ*^2^ | P |
| Treatment | 2 | 1.31 | 0.52 |  | 5.33 | 0.07 |  | 2.61 | 0.27 |  | 7.34 | **0.02** |  | 0.22 | 0.89 |
| Latitude | 1 | 1.02 | 0.31 |  | 3.11 | 0.08 |  | 1.81 | 0.18 |  | 0.02 | 0.89 |  | 8.64 | **0.003** |
| Treatment*Latitude | 2 | 0.97 | 0.61 |  | 8.74 | **0.01** |  | 0.09 | 0.95 |  | 3.41 | 0.18 |  | 0.18 | 0.92 |
| Total | 5 |  |  |  |  |  |  |  |  |  |  |  |  |  |  |
|  | **(f) Carbon rhizomes** | | |  | **(g) C:N ratio rhizomes** | | | **(h) Nitrogen leaves** | | | **(i) Carbon leaves** | |  | **(j) C:N ratio leaves** | |
| **Source of variation** | *df* | *χ*^2^ | P |  | *χ*^2^ | P |  | *χ*^2^ | P |  | *χ*^2^ | P |  | *χ*^2^ | P |
| Treatment | 2 | 1.06 | 0.59 |  | 1.15 | 0.56 |  | 8.20 | **0.02** |  | 3.22 | 0.20 |  | 8.58 | **0.01** |
| Latitude | 1 | 4.44 | **0.03** |  | 4.95 | **0.03** |  | 6.02 | **0.01** |  | 0.31 | 0.58 |  | 7.43 | **0.006** |
| Treatment*Latitude | 2 | 0.48 | 0.79 |  | 0.31 | 0.86 |  | 7.92 | **0.02** |  | 0.72 | 0.70 |  | 9.05 | **0.01** |
| Total | 5 |  |  |  |  |  |  |  |  |  |  |  |  |  |  |

**Table S2.** Results of linear mixed models testing for differences between treatments and time on different traits of *Posidonia australis* between austral summer 2015 and winter 2016 at the northernmost range edge of the seagrass*,* in shoots subjected to simulated herbivory treatments: control, low (10%) and high (80%) levels. Plot was included in the model as a random factor. Text in bold indicates significant (*p* < 0.05) differences. NSC = Total non-structural carbohydrates.

|  |  | | | | |  |  |  |  |  |  |  |  |  |  |
| --- | --- | --- | --- | --- | --- | --- | --- | --- | --- | --- | --- | --- | --- | --- | --- |
|  | **(a) Leaf growth (g DW shoot ^-1^ day^-1^)** | | | | **(b) NSC rhizomes** | |  | **(c) Phenolics leaves** | | | **(d) Double shoots** | |  | **(e) Nitrogen rhizomes** | |
| **Source** | df | ***χ*^2^** | P |  | ***χ*^2^** | P |  | ***χ*^2^** | P |  | ***χ*^2^** | P |  | ***χ*^2^** | P |
| Treatment | 2 | 6.11 | **0.047** |  | 0.14 | 0.93 |  | 0.52 | 0.77 |  | 1.47 | 0.48 |  | 0.02 | 0.99 |
| Time | 1 | 7.21 | **0.007** |  | 17.62 | **<0.001** |  | 3.50 | 0.06 |  | 6.41 | **0.01** |  | 0.54 | 0.46 |
| Treatment*Time | 2 | 1.37 | 0.505 |  | 5.62 | 0.06 |  | 4.62 | 0.10 |  | 2.00 | 0.37 |  | 0.91 | 0.64 |
| Total | 5 |  |  |  |  |  |  |  |  |  |  |  |  |  |  |
|  | **(f) Carbon rhizomes** | | |  | **(g) C:N ratio rhizomes** | | | **(h) Nitrogen leaves** | | | **(i) Carbon leaves** | |  | **(j) C:N ratio leaves** | |
| **Source of variation** | *df* | ***χ*^2^** | P |  | ***χ*^2^** | P |  | ***χ*^2^** | P |  | ***χ*^2^** | P |  |  |  |
| Treatment | 2 | 4.88 | 0.09 |  | 0.41 | 0.82 |  | 2.10 | 0.35 |  | 5.99 | **0.0499** |  | 1.67 | 0.44 |
| Time | 1 | 5.4 | **0.02** |  | 1.12 | 0.29 |  | 5.41 | **0.02** |  | 1.89 | 0.17 |  | 7.32 | **0.007** |
| Treatment*Time | 2 | 0.18 | 0.91 |  | 1.28 | 0.53 |  | 1.25 | 0.53 |  | 0.05 | 0.97 |  | 1.47 | 0.48 |
| Total | 5 |  |  |  |  |  |  |  |  |  |  |  |  |  |  |

**Table S3.** Results of linear mixed models testing for differences between treatments and latitude on different traits of *Posidonia australis* shoots subjected to simulated herbivory treatments: control, low (10%) and high (80%) levels. Location and Plot were included in the model as random factors.

|  |  |  |  |  |  |  |  |  |  |
| --- | --- | --- | --- | --- | --- | --- | --- | --- | --- |
|  |  | **Productivity** | |  |  |  |  |  |  |
|  |  | **(a) g DW m^2^ day^-1^** | |  | **(b) cm^2^ shoot^-1^ day^-1^** | |  | **(c) cm^2^ m^2^ day^-1^** | |
| **Source of variation** | *df* | ***χ*^2^** | P |  | ***χ*^2^** | P |  | ***χ*^2^** | P |
| Treatment | 2 | 1.30 | 0.52 |  | 0.65 | 0.72 |  | 0.28 | 0.87 |
| Latitude | 1 | 0.08 | 0.78 |  | 0.31 | 0.58 |  | 0.01 | 0.92 |
| Treatment*Latitude | 2 | 2.21 | 0.33 |  | 0.43 | 0.81 |  | 1.09 | 0.58 |
| Total | 5 |  |  |  |  |  |  |  |  |

**Table S4.** Results of linear mixed models testing for differences between treatments and time on *Posidonia australis* leaf growth measured as cm^2^ shoot^-1^ day^-1^ between austral summer 2015 and winter 2016 at the northernmost range edge of the seagrass*,* in shoots subjected to simulated herbivory treatments: control, low (10%) and high (80%) levels. Plot was included in the model as a random factor. Text in bold indicates significant (*p* < 0.05) differences.

|  |  |  |  |
| --- | --- | --- | --- |
|  |  | **cm^2^ shoot^-1^ day^-1^** | |
| **Source of variation** | *df* | ***χ*^2^** | P |
| Treatment | 2 | 3.55 | 0.17 |
| Time | 1 | 0.12 | 0.75 |
| Treatment*Time | 2 | 0.78 | 0.68 |
| Total | 5 |  |  |

**
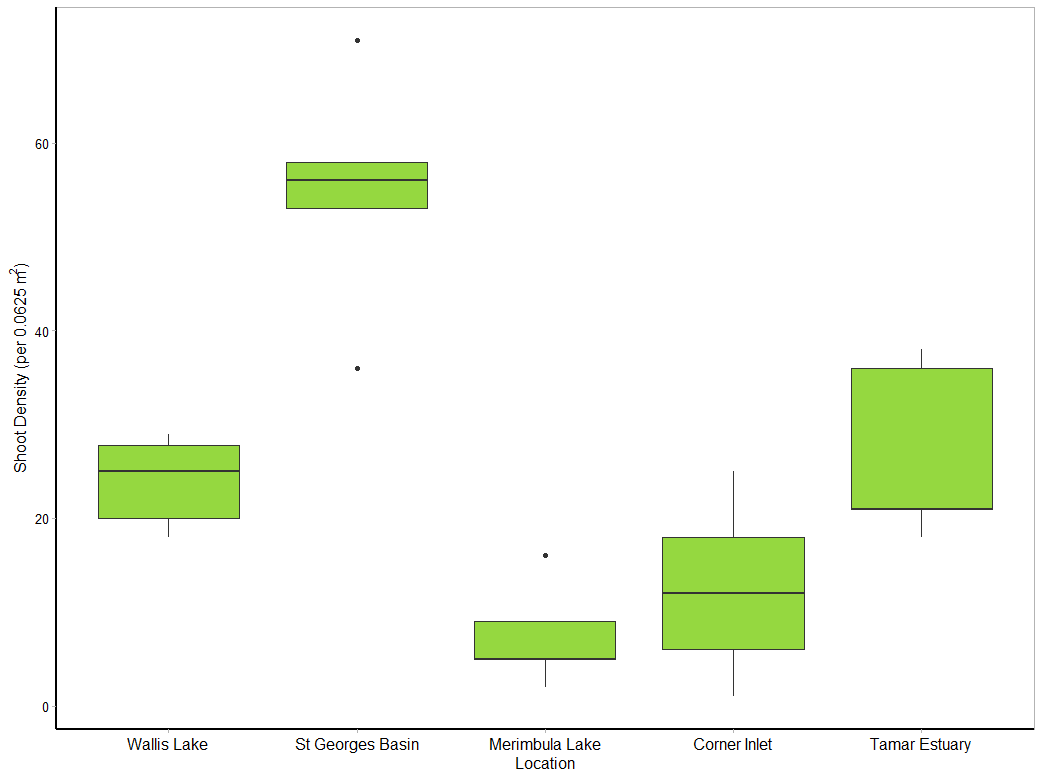
**

**Figure S1.** Seagrass shoot density (per 0.0625 m^2^) based on five replicate quadrats at each location, ordered north to south. Shoot density differed significantly among the five locations (F = 22.67, df = 4, *p* < 0.001), with Corner Inlet, Merimbula Lake, Tamar Estuary and Wallis Lake differing from St Georges Basin, and Tamar Estuary and Wallis Lake differing from Merimbula Lake.


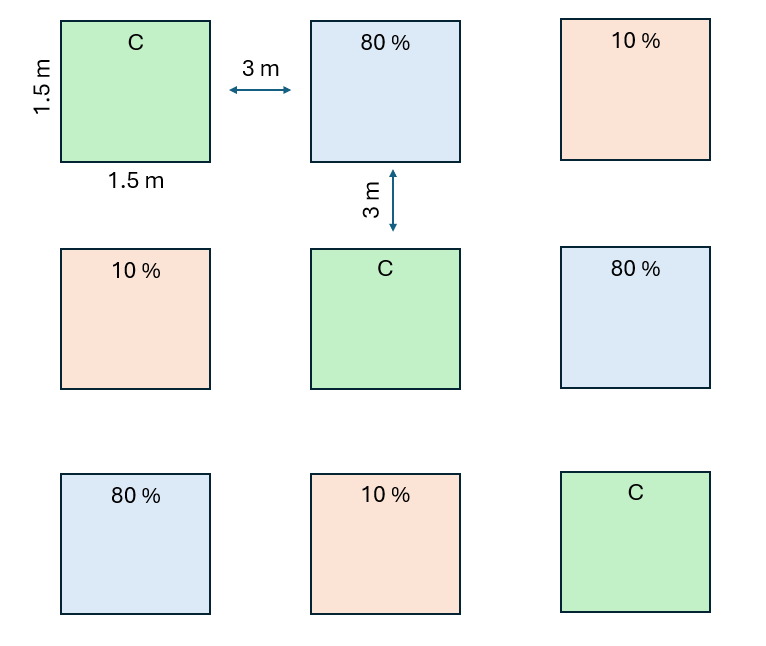


**Figure S2**. Example of spatial arrangements of the *Posidonia australis* simulated herbivory plots. C = control, shoots un-manipulated; 10% = removal of 10% of the leaf length, simulating low intensity herbivory and 80% = removal of 80% of the leaf length, simulating extensive one-off grazing events.


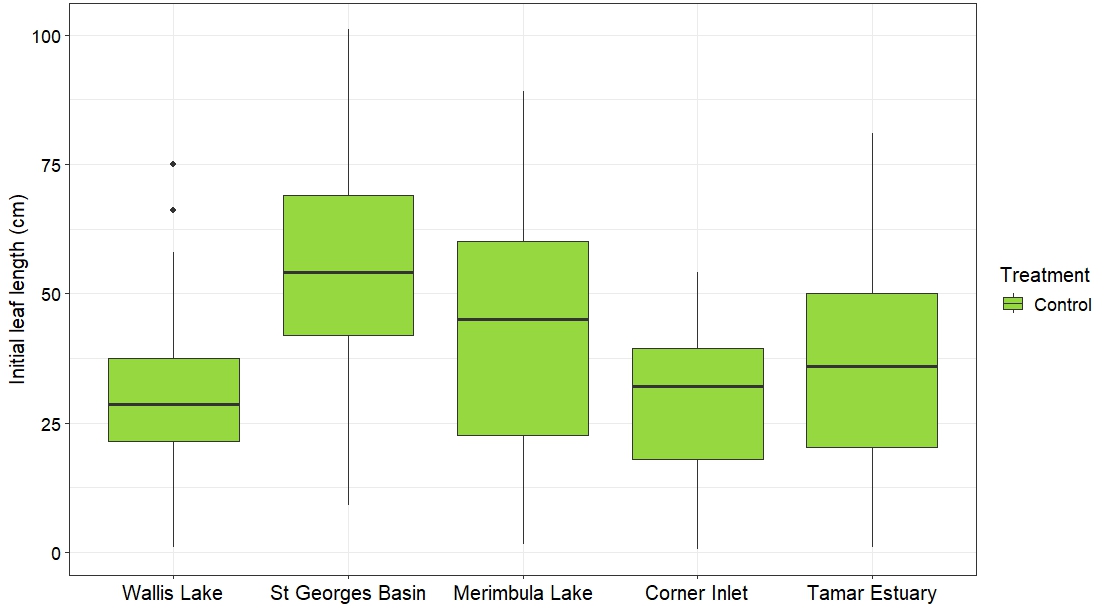


**Figure S3**. Initial leaf lengths of *P. australis* in control plots before the beginning of the experiment. Sites are ordered north to south by latitude, N = 79 – 118 leaves per location.

**
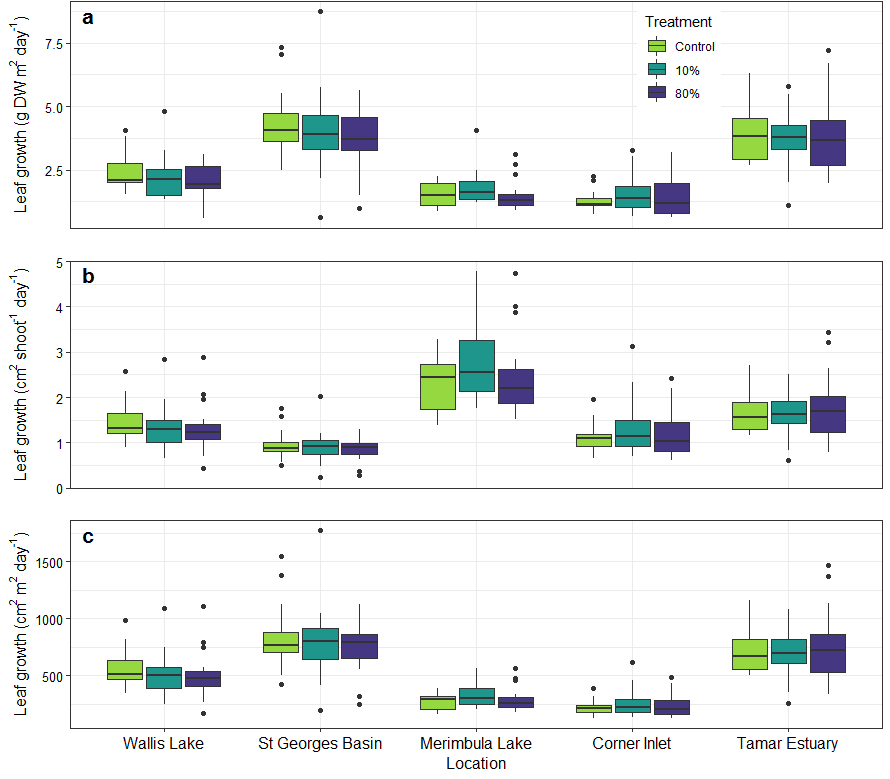
**

**Figure S4.** Leaf growth of *P. australis* shoots subjected to simulated herbivory treatments: control (C), low (10%) and high (80%) intensity, measured in (a) g DW m^2^ day^-1^ (b) cm^2^ shoot^-1^ day^-1^ (c) cm^2^ m^2^ day^-1^. Sites are ordered north to south by latitude, n = 21 – 30 shoots per treatment per sampling time for growth. DW = dry weight.


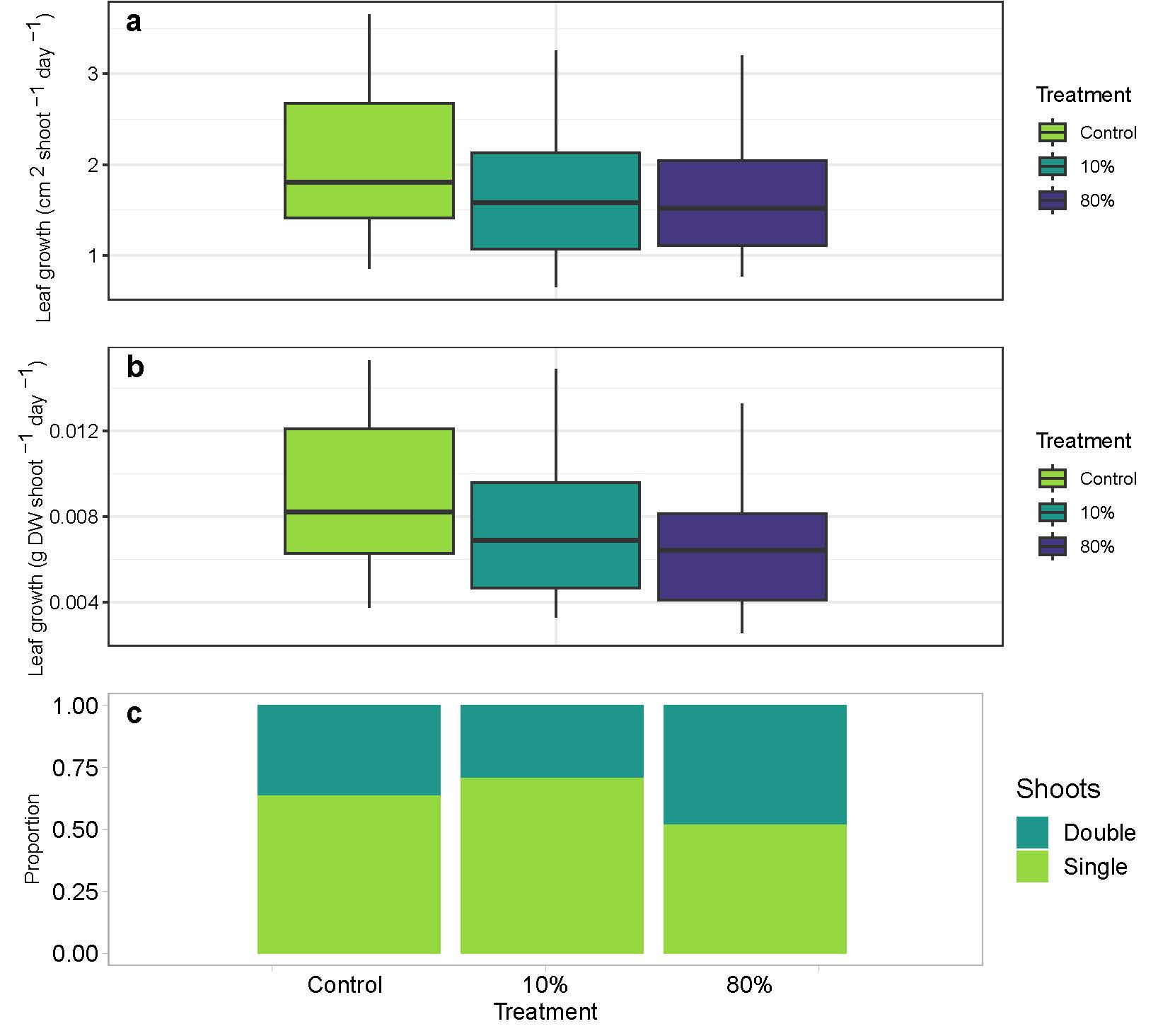


**Figure S5.** Leaf growth in (a) g DW shoot^-1^ day^-1^ in (b) cm^2^ shoot^-1^ day^-1^ and (c) proportion of shoots with additional growth modules (double shoots) of *P. australis* shoots subjected to repeated simulated herbivory treatments: control (C), low (10%) and high (80%) intensity. Maintenance clipping was carried out every 3 weeks between August 2016 and November 2016 at the northernmost site Wallis Lake. n = 21 – 30 shoots per treatment.

**
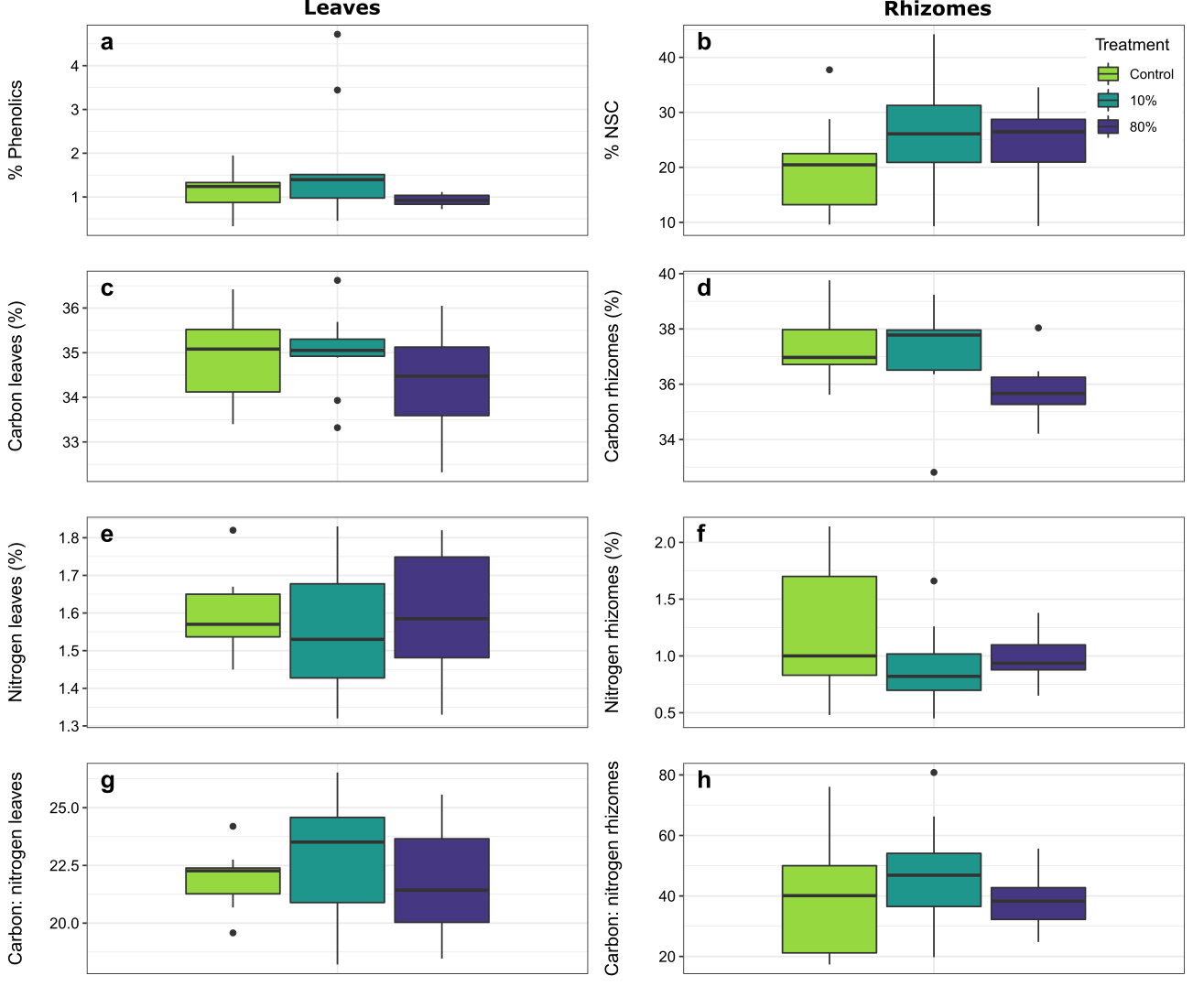
**

**Figure S6.** (a) Leaf phenolic content, (b) rhizome total non-structural carbohydrate (NSC) content, (c) leaf carbon content, (d) leaf nitrogen content, (e) rhizome carbon content, (f) rhizome nitrogen content, (g) leaf carbon: nitrogen ratio and (h) rhizome carbon: nitrogen ratio of P*. australis* shoots subjected to repeated simulated herbivory treatments: control (C), low (10%) and high (80%) intensity. Maintenance clipping was carried out every 3 weeks between August 2016 and November 2016 at the northernmost site Wallis Lake. n = 9 shoots per treatment for all traits.
